# The Genome of Deep-Sea Vent Chemolithoautotroph *Thiomicrospira crunogena* XCL-2

**DOI:** 10.1371/journal.pbio.0040383

**Published:** 2006-11-14

**Authors:** Kathleen M Scott, Stefan M Sievert, Fereniki N Abril, Lois A Ball, Chantell J Barrett, Rodrigo A Blake, Amanda J Boller, Patrick S. G Chain, Justine A Clark, Carisa R Davis, Chris Detter, Kimberly F Do, Kimberly P Dobrinski, Brandon I Faza, Kelly A Fitzpatrick, Sharyn K Freyermuth, Tara L Harmer, Loren J Hauser, Michael Hügler, Cheryl A Kerfeld, Martin G Klotz, William W Kong, Miriam Land, Alla Lapidus, Frank W Larimer, Dana L Longo, Susan Lucas, Stephanie A Malfatti, Steven E Massey, Darlene D Martin, Zoe McCuddin, Folker Meyer, Jessica L Moore, Luis H Ocampo, John H Paul, Ian T Paulsen, Douglas K Reep, Qinghu Ren, Rachel L Ross, Priscila Y Sato, Phaedra Thomas, Lance E Tinkham, Gary T Zeruth

**Affiliations:** 1 Biology Department, University of South Florida, Tampa, Florida, United States of America; 2 Biology Department, Woods Hole Oceanographic Institution, Woods Hole, Massachusetts, United States of America; 3 Lawrence Livermore National Laboratory, Livermore, California, United States of America; 4 Joint Genome Institute, Walnut Creek, California, United States of America; 5 Department of Biochemistry, University of Missouri, Columbia, Missouri, United States of America; 6 Division of Natural Sciences and Mathematics, The Richard Stockton College of New Jersey, Pomona, New Jersey, United States of America; 7 Oak Ridge National Laboratory, Oak Ridge, Tennessee, United States of America; 8 Molecular Biology Institute, University of California Los Angeles, Los Angeles, California, United States of America; 9 University of Louisville, Louisville, Kentucky, United States of America; 10 The Monsanto Company, Ankeny, Iowa, United States of America; 11 Center for Biotechnology, Bielefeld University, Bielefeld, Germany; 12 College of Marine Science, University of South Florida, St. Petersburg, Florida, United States of America; 13 The Institute for Genomic Research, Rockville, Maryland, United States of America; University of Arizona, United States of America

## Abstract

Presented here is the complete genome sequence of Thiomicrospira crunogena XCL-2, representative of ubiquitous chemolithoautotrophic sulfur-oxidizing bacteria isolated from deep-sea hydrothermal vents. This gammaproteobacterium has a single chromosome (2,427,734 base pairs), and its genome illustrates many of the adaptations that have enabled it to thrive at vents globally. It has 14 methyl-accepting chemotaxis protein genes, including four that may assist in positioning it in the redoxcline. A relative abundance of coding sequences (CDSs) encoding regulatory proteins likely control the expression of genes encoding carboxysomes, multiple dissolved inorganic nitrogen and phosphate transporters, as well as a phosphonate operon, which provide this species with a variety of options for acquiring these substrates from the environment. *Thiom. crunogena* XCL-2 is unusual among obligate sulfur-oxidizing bacteria in relying on the Sox system for the oxidation of reduced sulfur compounds. The genome has characteristics consistent with an obligately chemolithoautotrophic lifestyle, including few transporters predicted to have organic allocrits, and Calvin-Benson-Bassham cycle CDSs scattered throughout the genome.

## Abstract 2

Secrets of the deep are revealed from the genome sequence of*Thiomicrospira crunogena* XCL-2, a chemolithoautotrophic sulfur-oxidizing gammaproteobacterium isolated from deep-sea hydrothermal vents.

## Introduction

Deep-sea hydrothermal vent communities are sustained by prokaryotic chemolithoautotrophic primary producers that use the oxidation of electron donors available in hydrothermal fluid (H_2_, H_2_S, and Fe^+2^) to fuel carbon fixation [1–3]. The chemical and physical characteristics of their environment are dictated largely by the interaction of hydrothermal fluid and bottom water. When warm, reductant- and CO_2_-rich hydrothermal fluid is emitted from fissures in the basalt crust, it creates eddies as it mixes with cold, oxic bottom water. As a consequence, at areas where dilute hydrothermal fluid and seawater mix, a microorganism's habitat is erratic, oscillating from seconds to hours between dominance by hydrothermal fluid (warm; anoxic; abundant electron donors; 0.02 to >1 mM CO_2_) and bottom water (2 °C; oxic; 0.02 mM CO_2_) [4,5].

Common chemolithoautotrophic isolates from these “mixing zones” from hydrothermal vents include members of the genus *Thiomicrospira,* a group that originally included all marine, spiral-shaped sulfur-oxidizing bacteria. Subsequent analyses of 16S rDNA sequences have revealed the polyphyletic nature of this group; members of *Thiomicrospira* are distributed among the gamma and epsilon classes of the Proteobacteria. *Thiomicrospira crunogena,* a member of the cluster of *Thiomicrospira*s in the gamma class, was originally isolated from the East Pacific Rise [6]. Subsequently, *Thiom. crunogena* strains were cultivated or detected with molecular methods from deep-sea vents in both the Pacific and Atlantic, indicating a global distribution for this phylotype [7]. Molecular methods in combination with cultivation further confirmed the ecological importance of *Thiom. crunogena* and closely related species at deep-sea and shallow-water hydrothermal vents [8,9].

To provide the energy necessary for carbon fixation and cell maintenance, *Thiom. crunogena* XCL-2 and its close relatives Thiomicrospira spp. L-12 and MA-3 are capable of using hydrogen sulfide, thiosulfate, elemental sulfur, and sulfide minerals (e.g., pyrite and chalcopyrite) as electron donors; the only electron acceptor they can use is oxygen [6,10–12].

Given its temporally variable habitat, *Thiom. crunogena* XCL-2 is likely adapted to cope with oscillations in the availability of the inorganic nutrients necessary for chemolithoautotrophic growth. One critical adaptation in this habitat is its carbon-concentrating mechanism [13,14]. This species is capable of rapid growth in the presence of low concentrations of dissolved inorganic carbon, due to an increase in cellular affinity for both HCO_3_^−^ and CO_2_ under low-CO_2_ conditions [14]. The ability to grow under low-CO_2_ conditions is likely an advantage when the habitat is dominated by relatively low-CO_2_ seawater. Further adaptations in nutrient acquisition and microhabitat sensing are likely to be present in this organism.

*Thiom. crunogena* XCL-2 [15] is the first deep-sea autotrophic hydrothermal vent bacterium to have its genome completely sequenced and annotated. Many other autotrophic bacterial genomes have been examined previously, including several species of cyanobacteria (e.g., [16,17]), nitrifiers [18], purple nonsulfur [19], and green sulfur [20] photosynthetic bacteria, as well as an obligately chemolithoautotrophic sulfur-oxidizer [21] and a hydrogen-oxidizer [22]. These genomes have provided insight into the evolution of autotrophy among four of the seven phyla of Bacteria known to have autotrophic members.

The genome of *Thiom. crunogena* XCL-2 was sequenced to illuminate the evolution and physiology of bacterial primary producers from hydrothermal vents and other extreme environments. It was of interest to determine whether any specific adaptations to thrive in an environment with extreme temporal and spatial gradients in habitat geochemistry would be apparent from the genome. It was predicted that comparing its genome both to the other members of the Gammaproteobacteria, many of which are pathogenic heterotrophs, and also to autotrophs from the Proteobacteria and other phyla, would provide insights into the evolution and physiology of autotrophs within the Gammaproteobacteria. Further, this genome provides a reference point for uncultivated (to date) chemoautotrophic sulfur-oxidizing gammaproteobacterial symbionts of various invertebrates.

## Results/Discussion

### Genome Structure

**Figure 1:**
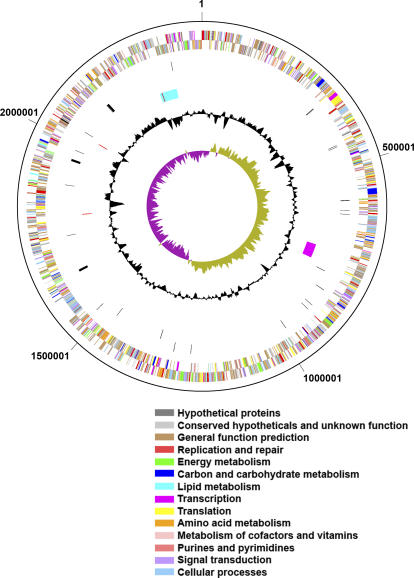
Circular Map of the *Thiom. crunogena* XCL-2 Genome The outer two rings (rings 1 and 2) are protein-encoding genes, which are color-coded according to COG category. Rings 3 and 4 are tRNA and rRNA genes. Ring 5 indicates the location of a prophage (magenta), phosphonate/heavy metal resistance island (cyan), and four insertion sequences (red; two insertion sequences at 2028543 and 2035034 are superimposed on this figure). The black circle indicates the deviation from the average %GC, and the purple and green circle is the GC skew (= [G − C]/[G + C]). Both the %GC and GC skew were calculated using a sliding window of 10,000 bp with a window step of 100.

**Figure 2:**
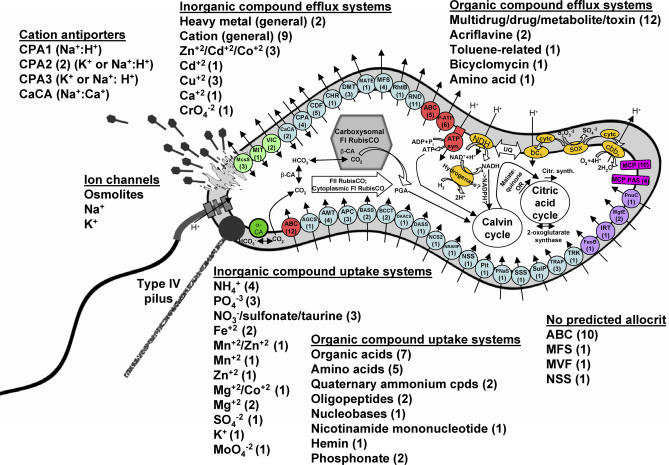
Cell Model for *Thiom. crunogena* XCL-2, with an Emphasis on Ultrastructure, Transport, Energy, Carbon Metabolism, and Chemotaxis Genes encoding virtually all of the steps for the synthesis of nucleotides and amino acids by canonical pathways are present in the bacterium, but are omitted here for simplicity. Electron transport components are yellow, and abbreviations are as follows: bc_1_, *bc_1_* complex; cbb_3_, *cbb_3_*-type cytochrome C oxidase; cytC, cytochrome C; NDH, NADH dehydrogenase; Sox, Sox system; UQ, ubiquinone. MCPs are fuchsia, as are MCPs with PAS domains or PAS folds. Influx and efflux transporter families with representatives in this genome are indicated on the figure, with the number of each type of transporter in parentheses. ATP-dependent transporters are red, secondary transporters are sky blue, ion channels are light green, and unclassified transporters are purple. Abbreviations for transporter families are as follows: ABC, ATP-binding cassette superfamily; AGCS, alanine or glycine:cation symporter family; AMT, ammonium transporter family; APC, amino acid-polyamine-organocation family; ATP syn, ATP synthetase; BASS, bile acid:Na^+^ symporter family; BCCT, betaine/carnitine/choline transporter family; CaCA, Ca^2+^:cation antiporter family; CDF, cation diffusion facilitator family; CHR, chromate ion transporter family; CPA, monovalent cation:proton antiporter-1, −2, and −3 families; DAACS, dicarboxylate/amino acid:cation symporter family; DASS, divalent anion:Na^+^ symporter family; DMT, drug/metabolite transporter superfamily; FeoB, ferrous iron uptake family; IRT, iron/lead transporter superfamily; MATE, multidrug/oligosaccharidyl-lipid/polysaccharide (MOP) flippase superfamily, MATE family; McsS, small conductance mechanosensitive ion channel family; MFS, major facilitator superfamily; MgtE, Mg^2+^ transporter-E family; MIT, CorA metal ion transporter family; NCS2, nucleobase:cation symporter-2 family; NRAMP, metal ion transporter family; NSS, neurotransmitter:sodium symporter family; P-ATP, P-type ATPase superfamily; Pit, inorganic phosphate transporter family; PNaS, phosphate:Na^+^ symporter family; PnuC, nicotamide mononucleotide uptake permease family; RhtB, resistance to homoserine/threonine family; RND, resistance-nodulation-cell division superfamily; SSS, solute:sodium symporter family; SulP, sulfate permease family; TRAP, tripartite ATP-independent periplasmic transporter family; TRK, K^+^ transporter family; VIC, voltage-gated ion channel superfamily.

*Thiom. crunogena* XCL-2 has a single chromosome consisting of 2.43 megabase pairs (Mbp), with a GC content of 43.1% and a high coding density (90.6 %; Figure 1). The GC skew shifts near the gene encoding the DnaA protein (located at “noon” on the circular map; *Tcr0001*), and thus the origin of replication is likely located nearby. One region with a deviation from the average %GC contains a phosphonate operon and has several other features consistent with its acquisition via horizontal gene transfer (see “Phosphorus Uptake” below). Many genes could be assigned a function with a high degree of confidence (Table 1), and a model for cell function based on these genes is presented (Figure 2).

**Table 1:**
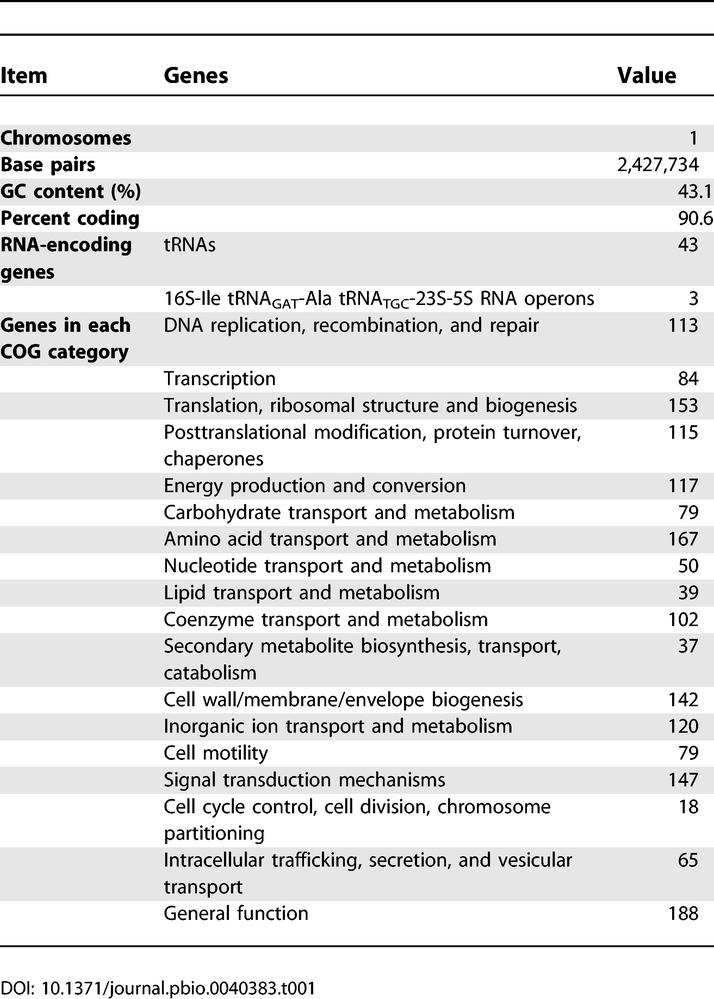
*Thiom. crunogena* XCL-2 Genome Summary

Three rRNA operons are present, and two of them, including their intergenic regions, are 100% identical. In the third rRNA operon, the 16S and 5S genes are 100% identical to the other two, but the 23S gene has a single substitution. The intergenic regions of this third operon also have several substitutions compared to the other two, with three substitutions between the tRNA-Ile-GAT and tRNA-Ala-TGC genes, six substitutions between the tRNA-Ala-TGC and 23S genes, and one substitution between the 23S and 5S genes. Having three rRNA operons may provide additional flexibility for rapid shifts in translation activity in response to a stochastic environment, and may contribute to this species' rapid doubling times [6]. Forty-three tRNA genes were identified by tRNA-scan SE [23] and the Search For RNAs program. An additional region of the chromosome was identified by Search For RNAs, the 3′ end of which is 57% identical with the sequence of the tRNA-Asn-GTT gene, but has a 47 nucleotide extension of the 5' end, and is a likely tRNA pseudogene.

### Prophage

**Figure 3:**
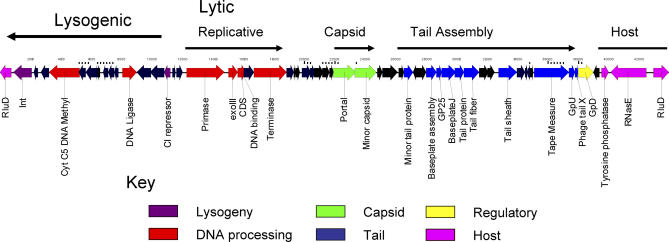
Prophage Genome within the *Thiom. crunogena* XCL-2 Genome Lysogenic and lytic genes are delineated, as are predicted gene functions.

A putative prophage genome was noted in the *Thiom. crunogea* chromosome. The putative prophage is 38,090 base pairs (bp) and contains 54 coding sequences (CDSs), 21 of which (38.9%) had significant similarity to genes in GenBank. The prophage genome begins with a tyrosine integrase *(Tcr0656)* and contains a *cI*-like repressor gene *(Tcr0666)*, features common to lambdoid prophages (Figure 3 [24]). These genes define a probable “lysogeny module” [25] and are in the opposite orientation from the rest of the phage genes (the replicative or “lytic module”).

The lytic half of the prophage genome encodes putative genes involved in DNA replication and phage assembly (Figure 3). Beginning with a putative DNA primase *(Tcr0668)* is a cluster of genes interpreted to represent an active or remnant DNA replication module (including an exonuclease of DNA polymerase, a hypothetical DNA binding protein, and a terminase large subunit: *Tcr0669, 0670,* and *0672*). Terminases serve to cut the phage DNA in genome-sized fragments prior to packaging. Beyond this are eight CDSs of unknown function, and then two CDSs involved in capsid assembly, including the portal protein *(Tcr0679)* and a minor capsid protein *(Tcr0680)* similar to GPC of λ. Portal proteins are ring-like structures in phage capsids through which the DNA enters the capsid during packaging [26]. In λ, the GPC protein is a peptidase (S49 family) that cleaves the capsid protein from a scaffolding protein involved in the capsid assembly process [27]. Although no major capsid protein is identifiable from bioinformatics, capsid proteins are often difficult to identify from sequence information in marine phages [28]. A cluster of *P2*-like putative tail assembly and structural genes follows the capsid assembly genes. The general organization of these genes (tail fiber, tail shaft and sheath, and tape measure; *Tcr0691; Tcr0690; Tcr0695;* and *Tcr0698*) is also *P2*-like [24]. The complexity of these genes (ten putative CDSs involved in tail assembly) and the strong identity score for a contractile tail sheath protein strongly argues that this prophage was a member of the Myoviridae, i.e., phages possessing a contractile tail. The final gene in the prophage-like sequence was similar to a phage late control protein D, gpD *(Tcr0700)*. In λ, gpD plays a role in the expansion of the capsid to accommodate the entire phage genome [29].

The high similarity of the CDSs to lambdoid (lysogeny and replication genes) and *P2*-like (tail module) temperate coliphages is surprising and unprecedented in marine prophage genomes [30]. A major frustration encountered in marine phage genomics is the low similarity of CDSs to anything in GenBank, making the interpretation of the biological function extremely difficult. The lambdoid siphophages are generally members of the Siphoviridae, whereas the *P2*-like phages are Myoviridae, which the *Thiom. crunogena* XCL-2 prophage is predicted to be. Such a mixed heritage is often the result of the modular evolution of phages. The general genomic organization of the *Thiom. crunogena* XCL-2 prophage-like element (integrase, repressor, DNA replicative genes, terminase, portal, capsid, tail genes) is common to several known prophages, including those of Staphylococcus aureus (i.e., ϕMu50B), Streptococcus pyogenes (prophages 370.3 and 370.2), and Streptococcus thermophilus (prophage O1205 [31]).

### Redox Substrate Metabolism and Electron Transport

Genes are present in this genome that encode all of the components essential to assemble a fully functional Sox system that performs sulfite-, thiosulfate-, sulfur-, and hydrogen sulfide–dependent cytochrome c reduction, namely, SoxXA *(Tcr0604* and *Tcr0601)*, SoxYZ (*Tcr0603* and *Tcr0602*), SoxB *(Tcr1549)*, and SoxCD *(Tcr0156*and *Tcr0157)* [32,33]. This well-characterized system for the oxidation of reduced sulfur compounds has been studied in facultatively chemolithoautotrophic, aerobic, thiosulfate-oxidizing alphaproteobacteria, including Paracococcus versutus GB17, *Thiobacillus versutus, Starkeya novella,* and Pseudoaminobacter salicylatoxidans ([32,34] and references therein). This model involves a periplasmic multienzyme complex that is capable of oxidizing various reduced sulfur compounds completely to sulfate. Genes encoding components of this complex have been identified, and it has further been shown that these so-called *“sox”* genes form extensive clusters in the genomes of the aforementioned bacteria. Essential components of the Sox system have also been identified in genomes of other bacteria known to be able to use reduced sulfur compounds as electron donors, resulting in the proposal that there might be a common mechanism for sulfur oxidation utilized by different bacteria [32,34]. Interestingly, *Thiom. crunogena* XCL-2 appears to be the first obligate chemolithoautotrophic sulfur-oxidizing bacterium to rely on the Sox system for oxidation of reduced sulfur compounds.

Genome analyses also reveal the presence of a putative sulfide:quinone reductase gene (*Tcr1170;* SQR). This enzyme is present in a number of phototrophic and chemotrophic bacteria and is best characterized from Rhodobacter capsulatus [35]. In this organism, it is located on the periplasmic surface of the cytoplasmic membrane, where it catalyzes the oxidation of sulfide to elemental sulfur, leading to the deposition of sulfur outside the cells. It seems reasonable to assume that SQR in *Thiom. crunogena* XCL-2 performs a similar function, explaining the deposition of sulfur outside the cell under certain conditions (e.g., low pH or oxygen [36]). The Sox system, on the other hand, is expected to result in the complete oxidation of sulfide to sulfate. Switching to the production of elemental sulfur rather than sulfate has the advantage that it prevents further acidification of the medium, which ultimately would result in cell lysis. An interesting question in this regard will be to determine how *Thiom. crunogena* XCL-2 remobilizes the sulfur globules. The dependence on the Sox system, and possibly SQR, for sulfur oxidation differs markedly from the obligately autotrophic sulfur-oxidizing betaproteobacterium *Thiobacillus denitrificans,* which has a multitude of pathways for sulfur oxidation, perhaps facilitating this organism's ability to grow under aerobic and anaerobic conditions [21].

In contrast to the arrangement in facultatively autotrophic sulfur-oxidizers [34], the *sox* components in *Thiom. crunogena* XCL-2 are not organized in a single cluster, but in different parts of this genome: *soxXYZA, soxB,* and *soxCD*. In particular, the isolated location of *soxB* relative to other *sox* genes has not been observed in any other sulfur-oxidizing organisms. The components of the Sox system that form tight interactions in vivo are collocated in apparent operons (SoxXYZA and SoxCD [37]), which is consistent with the “molarity model” for operon function (reviewed in [38]), in which cotranslation from a single mRNA facilitates interactions between tightly interacting proteins, and perhaps correct folding. Perhaps for obligate chemolithotrophs like *Thiom. crunogena* XCL-2 that do not have multiple sulfur oxidation systems, in which *sox* gene expression is presumably constitutive and not subject to complex regulation [39], *sox* gene organization into a single operon may not be strongly evolutionarily selected. Alternatively, the *Thiom. crunogena* XCL-2 *sox* genes may not be constitutively expressed, and may instead function as a regulon.

**Figure 4:**
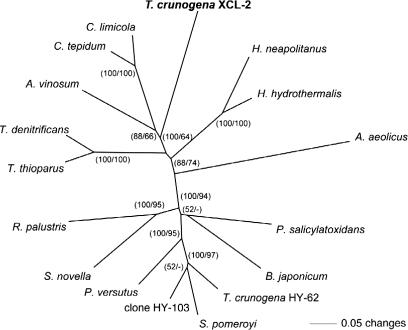
Phylogenetic Relationship of *Thiom. crunogena* XCL-2 SoxB to Sequences of Selected Bacteria Sequences were aligned using the program package MacVector. Neighbor-joining and parsimony trees based on the predicted amino acid sequences were calculated using PAUP 4.0b10 [113]. Bootstrap values (1,000 replicates) are given for the neighbor-joining (first value) and parsimony analyses (second value).

The confirmation of the presence of a *soxB* gene in *Thiom. crunogena* XCL-2 is particularly interesting, as it is a departure from previous studies with close relatives. Attempts to PCR-amplify *soxB* from *Thiom. crunogena* ATCC 700270^T^ and *Thiom. pelophila* DSM 1534^T^ were unsuccessful [40]. In contrast, a newly isolated *Thiomicrospira* strain obtained from a hydrothermal vent in the North Fiji Basin, *Thiom. crunogena* HY-62, was positive, with phylogenetic analyses further revealing that its *soxB* was most closely related those from Alphaproteobacteria, such as Silicibacter pomeroyi [40]. The *soxB* gene from *Thiom. crunogena* XCL-2 falls into a cluster containing the green-sulfur bacterium *Chlorobium* and the purple sulfur gammaproteobacterium *Allochromatium vinosum,* and separate from the cluster containing *soxB* from *Si. pomeroyi* and *Thiom. crunogena* HY-62 (Figure 4). This either indicates that *Thiom. crunogena* XCL-2 has obtained its *soxB* gene through lateral gene transfer from different organisms, or that the originally described *soxB* gene in *Thiom. crunogena* HY-62 was derived from a contaminant. The fact that both *soxA* and *soxX* from *Thiom. crunogena* XCL-2 also group closely with their respective homologs from *Chlorobium* spp argues for the latter (unpublished data). Also, the negative result for the two other *Thiomicrospira* strains is difficult to explain in light of the observation that sulfur oxidation in *Thiom. crunogena* XCL-2 appears to be dependent on a functional Sox system. It is possible that *Thiom. crunogena* ATCC 700270^T^ and *Thiom. pelophila* DSM 1543^T^ also have *soxB* genes, but that the PCR primers did not target conserved regions of this gene.

Up to this point, obligate chemolithoautotrophic sulfur oxidizers were believed to use a pathway different from the Sox system, i.e., the SI4 pathway [41] or a pathway that represents basically a reversal of dissimilatory sulfate reduction, by utilizing the enzymes dissimilatory sulfite reductase, APS reductase, and ATP sulfurylase [42]. In this context, it is interesting to note that *Thiom. crunogena* also seems to lack enzymes for the assimilation of sulfate, i.e., ATP sulfurylase, APS kinase, PAPS reductase, and a sirohaem-containing sulfite reductase, indicating that it depends on reduced sulfur compounds for both dissimilation and assimilation. *Thiom. crunogena* XCL-2 apparently also lacks a sulfite:acceptor oxidoreductase (SorAB), an enzyme evolutionarily related to SoxCD that catalyzes the direct oxidation of sulfite to sulfate and that has a wide distribution among different sulfur-oxidizing bacteria (Figure S1). The presence of the Sox system and the dependence on it in an obligate chemolithoautotroph also raises the question of the origin of the Sox system. Possibly, this system first evolved in obligate autotrophs before it was transferred into facultative autotrophs. Alternatively, *Thiom. crunogena* XCL-2 might have secondarily lost its capability to grow heterotrophically.

Genes for Ni/Fe hydrogenase large and small subunits are present *(Tcr2037* and *Tcr2038)*, as well as all of the genes necessary for large subunit metal center assembly *(Tcr2035–6* and *Tcr2039–2043)* [43]. Their presence and organization into an apparent operon suggest that *Thiom. crunogena* XCL-2 could use H_2_ as an electron donor for growth, as its close relative *Hydrogenovibrio* does [44,45]. However, attempts to cultivate *Thiom. crunogena* with H_2_ as the sole electron donor have not been successful [46]. A requirement for reduced sulfur compounds, even when not used as the primary electron donor, is suggested by the absence of genes encoding the enzymes necessary for assimilatory sulfate reduction (APS reductase and ATP sulfurylase), which are necessary for cysteine synthesis in the absence of environmental sources of thiosulfate or sulfide. Alternatively, this hydrogenase could act as a reductant sink under periods of sulfur and oxygen scarcity, when starch degradation could be utilized to replenish ATP and other metabolite pools (see Central Carbon Metabolism, below).

The redox partner for the *Thiom. crunogena* XCL-2 hydrogenase is suggested by the structure of the small subunit, which has two domains. One domain is similar to other hydrogenase small subunits, whereas the other is similar to pyridine nucleotide-disulphide oxidoreductases and has both an FAD and NADH binding site. The presence of an NADH binding site suggests that the small subunit itself transfers electrons between H_2_ and NAD(H), unlike other soluble hydrogenases, in which this activity is mediated by separate “diaphorase” subunits [43], which *Thiom. crunogena* XCL-2 lacks. The small subunit does not have the twin arginine leader sequence that is found in periplasmic and membrane-associated hydrogenases [47], suggesting a cytoplasmic location for this enzyme.

All 14 genes for the subunits of an electrogenic NADH:ubiquinone oxidoreductase (NDH-1) are present *(Tcr0817–0830)* and are organized in an apparent operon, as in other proteobacteria [48,49]. A cluster of genes encoding an RNF-type NADH dehydrogenase, which is evolutionarily distinct from NDH-1 [50], is present in the *Thiom. crunogena* XCL-2 genome *(Tcr1031–1036)*, and may shuttle NADH-derived electrons to specific cellular processes (as in [51]).

In this species, ubiquinone ferries electrons between NADH dehydrogenase and the *bc1* complex; all genes are present for its synthesis, but not for menaquinone. Unlike most bacteria, *Thiom. crunogena* XCL-2 does not synthesize the isopentenyl diphosphate units that make up the lipid portion of ubiquinone via the deoxyxylulose 5-phosphate pathway. Instead, most of the genes of the mevalonate pathway (HMG-CoA synthase, *Tcr1719;* HMG-CoA reductase, *Tcr1717;* mevalonate kinase/phosphomevalonate kinase, *Tcr1732, Tcr1733;* and diphosphomevalonate decarboxylase, *Tcr1734* [52]) are present. The single “missing” gene, for acetyl-CoA acetyltransferase, may not be necessary, because HMG-CoA reductase may also catalyze this reaction as it does in Enterococcus faecalis [53]. Interestingly, the mevalonate pathway is found in Archaea and eukaryotes, and is common among Gram-positive bacteria [52,54]. Thus far, the only other proteobacterium to have this pathway is from the alpha class, Paracoccus zeaxanthinifaciens [55]. Examination of unpublished genome data from the Integrated Microbial Genomes Web page (http://img.jgi.doe.gov/), and queries of Genbank (http://www.ncbi.nlm.nih.gov/Genbank) did not uncover evidence for a complete set of genes for the mevalonate pathway in other proteobacteria.

The three components of the *bc1* complex are represented by three genes in an apparent operon, in the typical order (Rieske iron-sulfur subunit; cytochrome *b* subunit; cytochrome *c1* subunit; *Tcr0991–3* [49]).

Consistent with its microaerophilic lifestyle and inability to use nitrate as an electron acceptor [6], the only terminal oxidase present in the *Thiom. crunogena* XCL-2 genome is a *cbb_3_*-type cytochrome c oxidase *(Tcr1963–5)*. To date, Helicobacter pylori is the only other sequenced organism that has solely a *cbb_3_*-type oxidase, and this has been proposed to be an adaptation to growth under microaerophilic conditions [49], since *cbb_3_*-type oxidase has a higher affinity for oxygen than *aa_3_-*type oxidase does [56].

In searching for candidate cytochrome proteins that facilitate electron transfer between the Sox system and the *bc_1_* complex and *cbb_3_* cytochrome c oxidase, the genome was analyzed to identify genes that encode proteins with heme-coordinating motifs (CxxCH). This search yielded 28 putative heme-binding proteins (Table S1), compared to 54 identified in the genome of *Thiob. denitrificans* [21]. Thirteen of these genes encode proteins that were predicted to reside in the periplasm, two of which *(Tcr0628* and *Tcr0628)* were deemed particularly promising candidates as they met the following criteria: (1) they were not subunits of other cytochrome-containing systems, (2) they were small enough to serve as efficient electron shuttles, (3) they were characterized beyond the level of hypothetical or conserved hypothetical, and (4) they were present in *Thiob. denitrificans,* which also has both a Sox system as well as *cbb_3_* cytochrome c oxidase, and had not been implicated in other cellular functions in this organism. *Tcr0628* and *Tcr0629* both belong to the COG2863 family of cytochrome c553, which are involved in major catabolic pathways in numerous proteobacteria. Interestingly, genes *Tcr0628* and *Tcr0629,* which are separated by a 147-bp spacer that includes a Shine-Delgarno sequence, are highly likely paralogs, and a nearly identical gene tandem was also identified in the genome of *Thiob. denitrificans (Tbd2026* and *Tbd2027)*. A recent comprehensive phylogenetic analysis of the cytochrome c553 proteins, including the mono-heme cytochromes from *Thiom. crunogena* and *Thiob. denitrificans,* revealed existence of a large protein superfamily that also includes proteins in the COG4654 cytochrome c551/c552 protein family (M. G. Klotz and A. B. Hooper, unpublished data). In ammonia-oxidizing bacteria, representatives of this protein superfamily (*NE0102, Neut2204,* and *NmulA0344* in the COG4654 protein family; and *Noc0751, NE0736,* and *Neut1650* in the COG2863 protein family) are the key electron carriers that connect the *bc1* complex with complex IV as well as NO_x_-detoxifying reductases (i.e., NirK and NirS) and oxidases (i.e., cytochrome P460 and cytochrome c peroxidase) involved in nitrifier denitrification ([57] and references therein). In Epsilonproteobacteria, such as *He. pylori* and *He. hepaticus,* cytochromes in this family *(jhp1148* and *HH1517)* interact with the terminal cytochrome *cbb_3_* oxidase. Therefore, we propose that the expression products of genes *Tcr0628* and *Tcr0629* likely represent the electronic link between the Sox system and the *bc1* complex and *cbb_3_* cytochrome c oxidase in *Thiom. crunogena*. It appears worthwhile to investigate experimentally whether the small difference in sequence between these two genes reflects an adaptation to binding to interaction partners with sites of different redox potential, namely cytochrome c_1_ in the *bc1* complex and cytochrome FixP (subunit III) in *cbb_3_* cytochrome c oxidase.

Given the presence of these electron transport complexes and electron carriers, a model for electron transport chain function is presented here (Figure 2). When thiosulfate or sulfide are acting as the electron donor, the Sox system will introduce electrons into the electron transport chain at the level of cytochrome c [32]. Most will be oxidized by the *cbb_3_*-type cytochrome c oxidase to create a proton potential. Some of the cytochrome c electrons will be used for reverse electron transport to ubiquinone and NAD^+^ by the *bc1* complex and NADH:ubiquinone oxidoreductase. The NADH created by reverse electron transport must contribute to the cellular NADPH pool for use in biosynthetic pathways. No apparent ortholog of either a membrane-associated [58] or soluble [59] transhydrogenase is present. A gene encoding a NAD^+^ kinase is present *(Tcr1633)*, and it is possible that it is also capable of phosphorylating NADH, as some other bacterial NAD^+^ kinases are [60].

### Transporters and Nutrient Uptake

**Figure 5:**
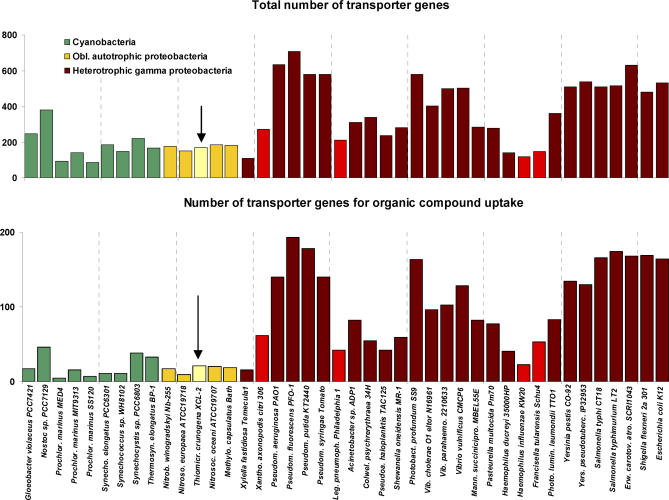
Transporter Gene Frequencies within the Genomes of *Thiom. crunogena* XCL-2 (Arrow) and Other Proteobacteria *Nitrob. winogradskyi (Nitrobacter winogradskyi)* is an alphaproteobacterium, *Nitros. europaea (Nitrosomonas europaea)* is a betaproteobacterium, and *Nitrosoc. oceani (Nitrosococcus oceani)* and *Methylo. capsulatus (Methylococcus capsulatus)* are gammaproteobacteria. Bars for intracellular pathogens are lighter red than the other heterotrophic gammaproteobacteria.

One hundred sixty-nine transporter genes from 40 families are present in the *Thiom. crunogena* XCL-2 genome (Figure 5), comprising 7.7% of the CDSs. This low frequency of transporter genes is similar to other obligately autotrophic proteobacteria and cyanobacteria as well as intracellular pathogenic bacteria such as *Xanthomonas axonopodis, Legionella pneumophila, Haemophilus influenzae,* and Francisella tularensis (Figure 5 [61,62]). Most heterotrophic gammaproteobacteria have higher transporter gene frequencies, up to 14.1% (Figure 5), which likely function to assist in the uptake of multiple organic carbon and energy sources, as suggested when transporters for sugars, amino acids and other organic acids, nucleotides, and cofactors were tallied (Figure 5).

### Carbon Dioxide Uptake and Fixation

*Thiom. crunogena* XCL-2, like many species of cyanobacteria [63], has a carbon-concentrating mechanism, in which active dissolved inorganic carbon uptake generates intracellular concentrations that are as much as 100× higher than extracellular [14]. No apparent homologs of any of the cyanobacterial bicarbonate or carbon dioxide uptake systems are present in this genome. *Thiom. crunogena* XCL-2 likely recruited bicarbonate and perhaps carbon dioxide transporters from transporter lineages evolutionarily distinct from those utilized by cyanobacteria. Three carbonic anhydrase genes are present (one α-class: *Tcr1545;* and two β-class: *Tcr0421* and *Tcr0841* [64–66]), one of which (α-class) is predicted to be periplasmic and membrane-associated, and may keep the periplasmic dissolved inorganic carbon pool at chemical equilibrium despite selective uptake of carbon dioxide or bicarbonate. One β-class enzyme gene is located near the gene for a form II RubisCO (see below) and may be co-expressed with it when the cells are grown under high-CO_2_ conditions. The other β-class (formerly ɛ-class; [66]) carbonic anhydrase is a member of a carboxysome operon and likely functions in this organism's carbon-concentrating mechanism. Unlike many other bacteria [67], the gene encoding the sole SulP-type ion transporter *(Tcr1533)* does not have a carbonic anhydrase gene adjacent to it.

The genes encoding the enzymes of the Calvin-Benson-Bassham (CBB) cycle are all present. Three ribulose 1,5-bisphosphate carboxylase/oxygenase (RubisCO) enzymes are encoded in the genome: two form I (FI) RubisCOs *(Tcr0427–8* and *Tcr0838–9)* and one form II (FII) RubisCO *(Tcr0424)*. The two FI RubisCO large subunit genes are quite similar to each other, with gene products that are 80% identical at the amino acid level. The FII RubisCO shares only 30% identity in amino acid sequence with both FI enzymes. The operon structure for each of these genes is similar to Hydrogenovibrio marinus [68]: one FI operon includes RubisCO structural genes *(cbbL* and *cbbS)* followed by genes encoding proteins believed to be important in RubisCO assembly *(cbbO* and *cbbQ; Tcr429–30)* [69,70]. The other FI operon is part of an α-type carboxysome operon *(Tcr0840–6)* [71] that includes carboxysome shell protein genes *csoS1, csoS2,* and *csoS3* (encoding a β-class carbonic anhydrase [65,66]). In the FII RubisCO operon, *cbbM* (encoding FII RubisCO) is followed by *cbbO* and *cbbQ* genes, which in turn are followed by a gene encoding a β-class carbonic anhydrase (*Tcr0421–3)* [64]. Differing from *Hy. marinus,* the noncarboxysomal FI and FII RubisCO operons are juxtaposed and divergently transcribed, with two genes encoding LysR-type regulatory proteins between them *(Tcr0425–6)*.

The genes encoding the other enzymes of the CBB cycle are scattered in the *Thiom. crunogena* XCL-2 genome, as in *Hy. marinus* [68]. This differs from facultative autotrophic proteobacteria, in which these genes are often clustered together and co-regulated [72–74]. Based on data from dedicated studies of CBB operons from a few model organisms, it has been suggested that obligate autotrophs like *Hy. marinus* do not have CBB cycle genes organized into an apparent operon, because these genes are presumably constitutively expressed and therefore do not need to be coordinately repressed [68].

Experimental evidence suggests that the CBB cycle is constitutively expressed in *Thiom. crunogena* XCL-2. This species cannot grow chemoorganoheterotrophically with acetate, glucose, or yeast extract as the carbon and energy source ([10]; Table S2). When grown in the presence of thiosulfate and dissolved inorganic carbon, RubisCO activities were high both in the presence and absence of these organic carbon sources in the growth medium (Table S3).

**Figure 6:**
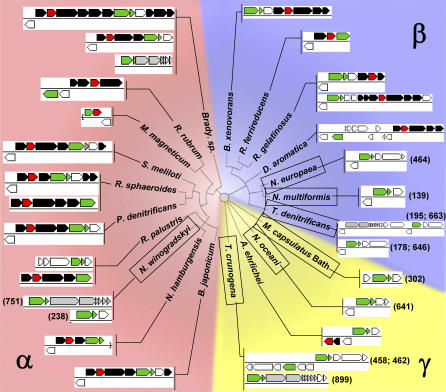
Calvin-Benson-Bassham Cycle Gene Organization in Proteobacteria RubisCO genes *(cbbLS* and *cbbM)* are green, phosphoribulokinase genes *(cbbP)* are red, other genes encoding Calvin-Benson-Bassham cycle enzymes are black, and carboxysome structural genes are grey. For species in which *cbbP* is not near *cbbLS* or *cbbM,* the distance from the RubisCO gene to *cbbP* in kbp is indicated in parentheses. *Thiob. denitrificans* has two *cbbP* genes, so two distances are indicated for this species. Names of organisms that are unable to grow well as organoheterotrophs are boxed. Abbreviations and accession numbers for the 16S sequences used to construct the cladogram are as follows: *A. ehrlichei, Alkalilimnicola ehrlichei,* AF406554; Brady. sp., Bradyrhizobium sp., AF338169;*B. japonicum, Bradyrhizobium japonicum,* D13430; *B. xenovorans, Burkholderia xenovorans*, U86373; *D. aromatica, Dechloromonas aromatica,* AY032610; *M. magneticum, Magnetospirillum magneticum,* D17514; *M. capsulatus, Methylococcus capsulatus* BATH, AF331869; *N. hamburgensis, Nitrobacter hamburgensis,* L11663; *N. winogradskyi, Nitrobacter winogradskyi,* L11661; *N. oceani, Nitrosococcus oceani,* AF363287; *N. europaea, Nitrosomonas europaea,* BX321856; *N. multiformis, Nitrosospira multiformis,* L35509; *P. denitrificans, Paracoccus denitrificans,* X69159; *R. sphaeroides, Rhodobacter sphaeroides,* CP000144; *R. ferrireducens, Rhodoferax ferrireducens,* AF435948; *R. palustris, Rhodopseudomonas palustris,* NC 005296; *R. rubrum, Rhodospirillum rubrum,* D30778; *R. gelatinosus, Rubrivivax gelatinosus,* M60682; *S. meliloti, Sinorhizobium meliloti,* D14509; *T. denitrificans, Thiobacillus denitrificans,* AJ43144; *T. crunogena, Thiomicrospira crunogena,* AF064545. The cladogram was based on an alignment of 1,622 bp of the 16S rRNA genes, and is the most parsimonious tree (length 2,735) resulting from a heuristic search with 100 replicate random step-wise addition and TBR branch swapping (PAUP*4.0b10 [113]). Sequences were aligned using ClustalW [114], as implemented in BioEdit. Percent similarities and identities for *cbbL, cbbM,* and *cbbP* gene products, as well as gene locus tags, are provided as supporting information (Table S4).

Many sequenced genomes from autotrophic bacteria have recently become available and provide a unique opportunity to determine whether CBB gene organization differs among autotrophs based on their lifestyle. Indeed, for all obligate autotrophs, RubisCO genes are not located near the genes encoding the other enzymes of the CBB cycle (Figure 6; Table S4). For example, the distance on the chromosome of these organisms between the genes encoding the only two enzymes unique to the CBB cycle, RubisCO *(cbbLS* and/or *cbbM)* and phosphoribulokinase *(cbbP)*, ranges from 139–899 kilobase pairs (kbp) in Proteobacteria, and 151–3,206 kbp in the Cyanobacteria. In contrast, for most facultative autotrophs, *cbbP* and *cbbLS* and/or *cbbM* genes are near each other (Figure 6); in most cases, they appear to coexist in an operon. In the facultative autotroph *Rhodospirillum rubrum,* the *cbbM* and *cbbP* genes occupy adjacent, divergently transcribed operons *(cbbRM* and *cbbEFPT)*. However, these genes are coordinately regulated, since binding sites for the regulatory protein *cbbR* are present between the operons [75]; perhaps they are coordinately repressed by a repressor protein that binds there as well. The lack of CBB enzyme operons in obligate autotrophs from the Alpha-. Beta-, and Gammaproteobacteria, as well as the Cyanobacteria, may reflect a lack of selective pressure for these genes to be juxtaposed in their chromosomes for ease of coordinate repression during heterotrophic growth.

### Central Carbon Metabolism

**Figure 7:**
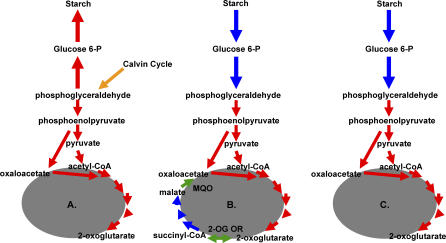
Models for Glycolysis, Gluconeogenesis, and the CAC in *Thiom. crunogena* XCL-2 Models for central carbon metabolism for cells under environmental conditions with (A) sufficient reduced sulfur and oxygen; (B) sulfide scarcity; and (C) oxygen scarcity; green arrows represent the two “non-canonical” CAC enzymes, 2-oxoglutarate oxidoreductase (2-OG OR) and malate: quinone oxidoreductase (MQO).

3-Phosphoglyceraldehyde generated by the Calvin-Benson-Bassham cycle enters the Embden-Meyerhof-Parnass pathway in the middle, and some carbon must be shunted in both directions to generate the carbon “backbones” for lipid, protein, nucleotide, and cell wall synthesis (Figure 7). All of the enzymes necessary to direct carbon from 3-phosphoglyceraldehyde to fructose-6-phosphate and glucose are encoded by this genome, as are all of the genes needed for starch synthesis. To convert fructose 1,6-bisphosphate to fructose 6-phosphate, either fructose bisphosphatase or phosphofructokinase could be used, as this genome encodes a reversible PP_i_-dependent phosphofructokinase *(Tcr1583)* [76,77]. This store of carbon could be sent back through glycolysis to generate metabolic intermediates to replenish levels of cellular reductant (see below). Genes encoding all of the enzymes necessary to convert 3-phosphoglyceraldehyde to phosphoenolpyruvate and pyruvate are present, and the pyruvate could enter the citric acid cycle (CAC) via pyruvate dehydrogenase, as genes encoding all three subunits of this complex are represented *(Tcr1001–3)* and activity could be measured with cell-free extracts of cultures grown in the presence and absence of glucose (M. Hügler and S. M. Sievert, unpublished data).

All of the genes necessary for an oxidative CAC are potentially present, as in some other obligate autotrophs and methanotrophs [18,78]. However, some exceptions from the canonical CAC enzymes seem to be present. The T. crunogena XCL-2 genome encodes neither a 2-oxoglutarate dehydrogenase nor a typical malate dehydrogenase, but it does have potential substitutions: a 2-oxoacid:acceptor oxidoreductase **(**α and β subunit genes in an apparent operon, *Tcr1709–10*)**,** and malate: quinone-oxidoreductase *(Tcr1873)*, as in *He. pylori* [79,80]. 2-Oxoacid:acceptor oxidoreductase is reversible, unlike 2-oxoglutarate dehydrogenase, which is solely oxidative [79,81]**.** An overall oxidative direction for the cycle is suggested by malate:quinone oxidoreductase. This membrane-associated enzyme donates the electrons from malate oxidation to the membrane quinone pool and is irreversible, unlike malate dehydrogenase, which donates electrons to NAD^+^ [80]. The 2-oxoacid:acceptor oxidoreductase shows high similarity to the well-characterized 2-oxoglutarate:acceptor oxidoreductase of Thauera aromatica [82], suggesting that it might catalyze the conversion 2-oxoglutrate rather than pyruvate as a substrate. However, cell-free extracts of cells grown autotrophically in the presence and absence of glucose have neither 2-oxoglutarate- nor pyruvate:acceptor oxidoreductase activity (M. Hügler and S. M. Sievert, unpublished data); thus, the CAC does not appear to be complete under these conditions.

A wishbone-shaped reductive citric acid pathway is suggested by this apparent inability to catalyze the interconversion of succinyl-CoA and 2-oxoglutarate. However, even though genes are present encoding most of the enzymes of the reductive arm of the reductive citric acid pathway, from oxaloacetate to succinyl CoA (phosphoenolpyruvate carboxylase, *Tcr1521;* fumarate hydratase, *Tcr1384;* succinate dehydrogenase/fumarate reductase, *Tcr2029–31;* succinyl-CoA synthetase; *Tcr1373–4*), the absence of malate dehydrogenase and malic enzyme genes, and the presence of a gene encoding malate:quinone-oxidoreductase (MQO) suggests a blockage of the reductive path as well.

A hypothesis for glycolysis/gluconeogenesis/CAC function is presented here to reconcile these observations (Figure 7). Under conditions in which reduced sulfur compounds and oxygen are sufficiently plentiful to provide cellular reductant and ATP for the Calvin cycle and other metabolic pathways, some carbon would be directed from glyceraldehyde 3-phosphate through gluconeogenesis to starch, whereas some would be directed to pyruvate and an incomplete CAC to meet the cell's requirements for 2-oxoglutarate, oxaloacetate, and other carbon skeletons. Succinyl-CoA synthesis may not be required, because in most bacteria [83], this genome encodes the enzymes of an alternative pathway for porphyrin synthesis via 5-amino levulinate (glutamyl-tRNA synthetase, *Tcr1216;* glutamyl tRNA reductase, *Tcr0390;* glutamate 1-semialdehyde 2,1 aminomutase; *Tcr0888*). Should environmental conditions shift to sulfide scarcity, cells could continue to generate ATP, carbon skeletons, and cellular reductant by hydrolyzing the starch and sending it through glycolysis and a full oxidative CAC. Should oxygen become scarce instead, cells could send carbon skeletons derived from starch through the incomplete CAC and oxidize excess NADH via the cytoplasmic Ni/Fe hydrogenase, which would also maintain a membrane proton potential via intracellular proton consumption. Clearly, the exact regulation of the CAC under different growth conditions promises to be an interesting topic for future research.

Genes encoding isocitrate lyase and malate synthase are missing, indicating the absence of a glyoxylate cycle, and consistent with this organism's inability to grow with acetate as the source of carbon (Table S2).

Nitrogen uptake and assimilation are described in Protocol S1 and Table S5.

### Phosphorus Uptake

**Figure 8:**
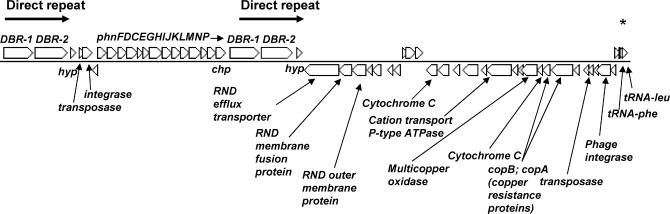
*Thiom. crunogena* XCL-2 Phosphonate Operon The *DBR-1* genes are identical to each other, as are the *DBR-2* genes. Gene abbreviations are: *chp*, conserved hypothetical protein; *DBR-1* and -*2*, DNA breaking-rejoining enzymes; *hyp*, hypothetical protein; *phnFDCEGHIJKLMNP*, phosphonate operon. An asterisk (*) marks the location of a region (within and upstream of tRNA-phe) with a high level of similarity to the 5′ ends of the two direct repeat sequences noted in the figure. The transposase and integrase are actually a single CDS separated by a frameshift.

T. crunogena XCL-2 has all of the genes for the low affinity PiT system *(Tcr0543–4)* and an operon encoding the high affinity Pst system for phosphate uptake *(Tcr0537–9)* [84]. *Thiom. crunogena* XCL-2 may also be able to use phosphonate as a phosphorus source, as it has an operon, *phnFDCEGHIJKLMNP (Tcr2078–90),* encoding phosphonate transporters and the enzymes necessary to cleave phosphorus-carbon bonds (Figure 8). This phosphonate operon is flanked on either side by large (>6,500 bp) 100% identical direct repeat elements. These elements encode three predicted CDSs *(Tcr2074–6* and *Tcr2091–3)*: a small hypothetical, and two large (>2,500 amino acids [aa] in length) CDSs with limited similarity to a phage-like integrase present in *Desulfuromonas acetoxidans,* including a domain involved in breaking and rejoining DNA (DBR-1 and DBR-2). It is interesting to note that two homologs found in the draft sequence of the high GC (~65%) Gammaproteobacterium Azotobacter vinelandii AvOP have a similar gene organization to the large putative integrases DBR-1/DBR-2. Directly downstream of the first copy of this large repeat element (and upstream of the phosphonate operon) lies another repeat, one of the four IS911-related IS3-family insertion sequences [85] present in this genome (Figure 1). Along with the presence of the transposase/integrase genes and the flanking large repeat element (likely an IS element), the strikingly different G+C of this entire region (39.6%) and the direct repeats (35.9%) compared to the genome average (43.1%) suggest that this region may have been acquired by horizontal gene transfer.

Interestingly, immediately downstream of this island lies another region of comparatively low G+C (39.6%) that encodes a number of products involved in metal resistance (e.g., copper transporters and oxidases, heavy metal efflux system). Directly downstream of this second island lies a phage integrase *(Tcr2121)* adjacent to two tRNAs, which are known to be common phage insertion sites. Strikingly, there is a high level of similarity between the 5′ region of the first tRNA—and its promoter region—and the 5′ regions of the large repeat elements, particularly the closest element (Figure 8). Taken together, it is proposed that this entire region has been horizontally acquired. Interestingly, it appears that the phosphonate operon from the marine cyanobacterium Trichodesmium erythraeum was also acquired by horizontal gene transfer [86]. Phylogenetic analyses reveal that the PhnJ protein of *Thiom. crunogena* XCL-2 falls into a cluster that, with the exception of *Tr. erythraeum,* contains sequences from gamma- and betaproteobacteria, with the sequence of *Thiob. denitrificans,* another sulfur-oxidizing bacterium, being the closest relative (Figure S2). The potential capability to use phosphonates, which constitute a substantial fraction of dissolved organic phosphorus [87], might provide *Thiom. crunogena* XCL-2 a competitive advantage in an environment that may periodically experience a scarcity of inorganic phosphorous. Any excess phosphate accumulated by *Thiom. crunogena* XCL-2 could be stored as polyphosphate granules, because polyphosphate kinase and exopolyphosphatase genes are present *(Tcr1891–2)*.

### Regulatory and Signaling Proteins

Despite its relative metabolic simplicity as an obligate autotroph, *Thiom. crunogena* XCL-2 allocates a substantial fraction of its protein-encoding genes (8.9%) to regulatory and signaling proteins (Table 2). In order to determine whether this was typical for a marine obligately chemolithoautotrophic gammaproteobacterium, the numbers of regulatory and signaling protein-encoding genes from this organism were compared to the only other such organism sequenced to date, Nitrosococcus oceani ATCC 19707 [88]. It was of interest to determine whether the differences in their habitats (*Thiom. crunogena:* attached, and inhabiting a stochastic hydrothermal vent environment, vs. N. oceani: planktonic, in a comparatively stable open-ocean habitat; [89]) would affect the sizes and compositions of their arsenals of regulatory and signaling proteins. Noteworthy differences between the two species include a high proportion of genes with EAL and GGDEF domains in *Thiom. crunogena* XCL-2 compared to N. oceani (Table 2). These proteins catalyze the hydrolysis and synthesis of cyclic diguanylate, suggesting the importance of this compound as an intracellular signaling molecule in *Thiom. crunogena* XCL-2 [90]. In some species the abundance of intracellular cyclic diguanylate dictates whether the cells will express genes that facilitate an attached vs. planktonic lifestyle [90]. Given that *Thiom. crunogena* was isolated by collecting scrapings from hydrothermal vent surfaces [6,15], perhaps cyclic diguanylate has a similar function in *Thiom. crunogena* as well.

**Table 2:**
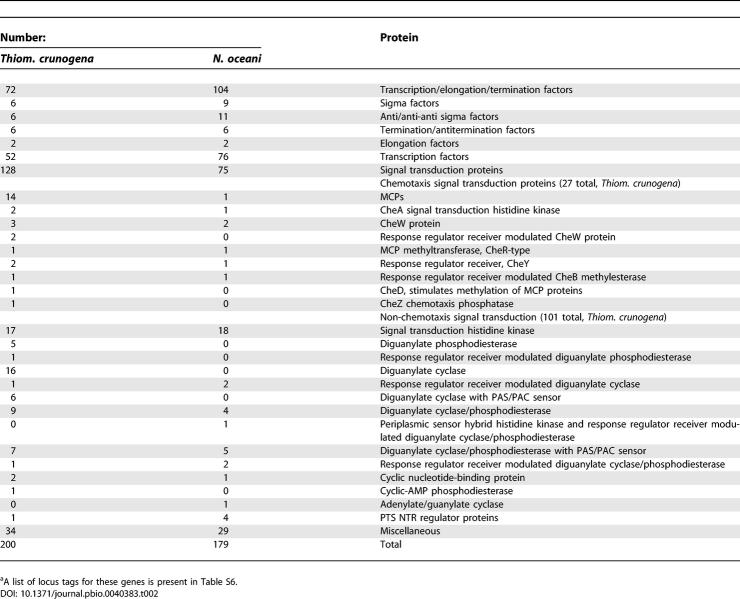
*Thiom. crunogena* XCL-2^a^ and *N. oceani* ATCC 19707 Regulatory and Signaling Proteins

Many of these EAL and GGDEF-domain proteins, and other predicted regulatory and signaling proteins, have PAS domains (Table 2; Table S6), which often function as redox and/or oxygen sensors by binding redox or oxygen-sensitive ligands (e.g., heme and FAD [91]). Twenty PAS-domain proteins predicted from *Thiom. crunogena* XCL-2′s genome sequence include four methyl-accepting chemotaxis proteins (MCPs) (see below), three signal transduction histidine kinases, six diguanylate cyclases, and seven diguanylate cyclase/phosphodiesterases. N. oceani has 14 predicted gene products with PAS/PAC domains; notable differences from *Thiom. crunogena* XCL-2 are an absence of PAS/PAC domain MCPs, and fewer PAS/PAC domain proteins involved in cyclic diguanylate metabolism (seven diguanylate cyclase/phosphodiesterases).

Despite its metabolic and morphological simplicity, *Thiom. crunogena* XCL-2 has almost as many genes encoding transcription factors (52) as the cyst and zoogloea-forming N. oceani does (76; Table 2 [89]). Indeed, most free-living bacteria have a considerably lower frequency of genes encoding regulatory and signaling proteins (5.6% in N. oceani [88]; 5%–6% in other species [19]). Other organisms with frequencies similar to *Thiom. crunogena* XCL-2 (8.6%) include the metabolically versatile Rhodopseudomonas palustris (9.3% [19]). Although *Thiom. crunogena* XCL-2 is not metabolically versatile, it has several apparent operons that encode aspects of its structure and metabolism that are likely to enhance growth under certain environmental conditions (e.g., carboxysomes, phosphonate metabolism, assimilatory nitrate reductase, and hydrogenase). Perhaps the relative abundance of regulatory and signaling protein-encoding genes in *Thiom. crunogena* XCL-2 is a reflection of the remarkable temporal and spatial heterogeneity of its hydrothermal vent habitat.

### Chemotaxis

Genes encoding the structural, regulatory, and assembly-related components of *Thiom. crunogena* XCL-2′s polar flagellae are organized into *flg (Tcr1464–77)* and *fla/fli/flh* clusters, similar to Vibrio spp. [92]. However, the *fla/fli/flh* cluster is split into two separate subclusters in *Thiom. crunogena* XCL-2 *(Tcr0739–47* and *Tcr1431–53).*

**Figure 9:**
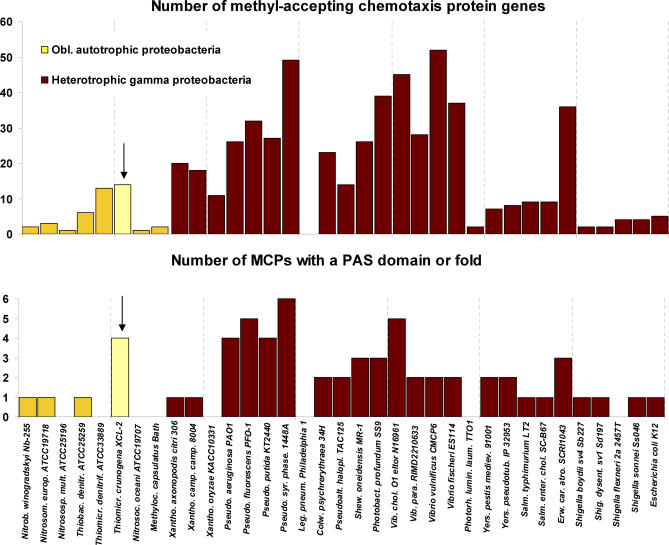
Numbers of MCP Genes in *Thiom. crunogena* XCL-2 and Other Proteobacteria *Thiom. crunogena* is marked with an arrow.

Fourteen genes encoding MCPs are scattered throughout the genome, which is on the low end of the range of MCP gene numbers found in the genomes of gammaproteobacteria. The function of MCPs is to act as nutrient and toxin sensors that communicate with the flagellar motor via the CheA and CheY proteins [93]. As each MCP is specific to a particular nutrient or toxin, it is not surprising that *Thiom. crunogena* XCL-2 has relatively few MCPs, because its nutritional needs as an autotroph are rather simple. Interestingly, however, the number of MCP genes is high for obligately autotrophic proteobacteria (Table 2; Figure 9), particularly with respect to those containing a PAS domain or fold (Figure 9). The relative abundance of MCPs in *Thiom. crunogena* XCL-2 may be an adaptation to the sharp chemical and redox gradients and temporal instability of *Thiom. crunogena* XCL-2′s hydrothermal vent habitat [4].

### Adhesion

A cluster of genes encoding pilin and the assembly and secretion machinery for type IV pili is present *(flp tadE cpaBCEF tadCBD; Tcr1722–30)*. In Actinobacillus actinomycetemcomitans and other organisms, these fimbrae mediate tight adherence to a variety of substrates [94]. *Thiom. crunogena* was originally isolated from a biofilm [6]. Adhesion within biofilms may be mediated by these fimbrae.

### Heavy Metal Resistance

Despite being cultivated from a habitat that is prone to elevated concentrations of toxic heavy metals including nickel, copper, cadmium, lead, and zinc [95,96], *Thiom. crunogena* XCL-2′s arsenal of heavy metal efflux transporter genes does not distinguish it from Escherichia coli and other Gammaproteobacteria. It has 11 sets of resistance-nodulation-cell division superfamily (RND)-type transporters, five cation diffusion facilitator family (CDF) transporters, and six P-type ATPases, far fewer than the metal-resistant Ralstonia metallidurans (20 RND, three CDF, and 20 P-type [97]), and lacking the arsenate, cadmium, and mercury detoxification systems present in the genome of hydrothermal vent heterotroph Idiomarina loihiensis [98]. To verify this surprising result, *Thiom. crunogena* XCL-2 was cultivated in the presence of heavy metal salts to determine its sensitivities to these compounds (Table 3). Indeed, *Thiom. crunogena* XCL-2 is not particularly resistant to heavy metals; instead, it is more sensitive to them than E. coli [99]. Similar results were found for hydrothermal vent archaea [100]; for these organisms, the addition of sulfide to the growth medium was found to enhance their growth in the presence of heavy metal salts, and it was suggested that, in situ at the vents, sulfide might “protect” microorganisms from heavy metals by complexing with metals or forming precipitates with them [100]. Potentially, this strategy is utilized by *Thiom. crunogena* XCL-2. Alternatively, hydrothermal fluid at its mesophilic habitat may be so dilute that heavy metal concentrations do not get high enough to necessitate extensive adaptations to detoxify them.

**Table 3:**
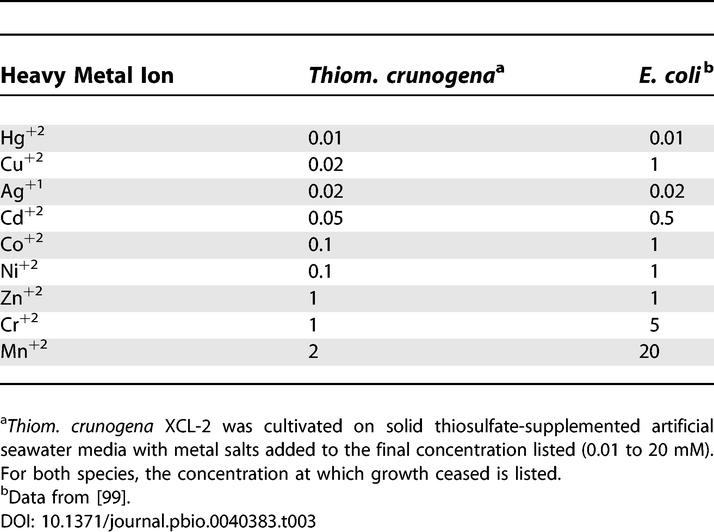
Growth-Inhibiting Concentrations (mM) of Heavy Metals for *Thiom. crunogena* XCL-2 and E. coli

### Conclusions

Many abilities are apparent from the genome of *Thiom. crunogena* XCL-2 that are likely to enable this organism to survive the spatially and temporally complex hydrothermal vent environment despite its simple, specialized metabolism. Instead of having multiple metabolic pathways, *Thiom. crunogena* XCL-2 appears to have multiple adaptations to obtain autotrophic substrates. Fourteen MCPs presumably guide it to microhabitats with characteristics favorable to its growth, and type IV pili may enable it to live an attached lifestyle once it finds these favorable conditions. A larger-than-expected arsenal of regulatory proteins may enable this organism to regulate multiple mechanisms for coping with variations in inorganic nutrient availability. Its three RubisCO genes, three carbonic anhydrase genes, and carbon-concentrating mechanism likely assist in coping with oscillations in environmental CO_2_ availability, while multiple ammonium transporters, nitrate reductase, low- and high-affinity phosphate uptake systems, and potential phosphonate use, may enable it to cope with uncertain supplies of these macronutrients.

In contrast, systems for energy generation are more limited, with only one, i.e., Sox, or possibly two, i.e., Sox plus SQR, systems for sulfur oxidation and a single low oxygen–adapted terminal oxidase (*cbb_3_*-type). Instead of having a branched electron transport chain with multiple inputs and outputs, this organism may use the four PAS-domain or -fold MCPs to guide it to a portion of the chemocline where its simple electron transport chain functions. It is worth noting, in this regard, that *Thiob. denitrificans,* which has several systems for sulfur oxidation, has fewer MCPs than *Thiom. crunogena* XCL-2 (Figure 9). Differential expression of portions of the CAC may enable it to survive periods of reduced sulfur or oxygen scarcity during its “transit” to more favorable microhabitats.

Up to this point, advances in our understanding of the biochemistry, genetics, and physiology of this bacterium have been hampered by a lack of a genetic system. The availability of the genome has provided an unprecedented view into the metabolic potential of this fascinating organism and an opportunity use genomics techniques to address the hypotheses mentioned here and others as more autotrophic genomes become available.

## Materials and Methods

### Library construction, sequencing, and sequence quality.

Three DNA libraries (with approximate insert sizes of 3, 7, and 35 kb) were sequenced using the whole-genome shotgun method as previously described [18]. Paired-end sequencing was performed at the Production Genomics Facility of the Joint Genome Institute (JGI), generating greater than 50,000 reads and resulting in approximately 13× depth of coverage. Approximately 400 additional finishing reads were sequenced to close gaps and address base quality issues. Assemblies were accomplished using the PHRED/PHRAP/CONSED suite [101–103], and gap closure, resolution of repetitive sequences, and sequence polishing were performed as previously described [18].

### Gene identification and annotation.

Two independent annotations were undertaken: one by the Genome Analysis and System Modeling Group of the Life Sciences Division of Oak Ridge National Laboratory (ORNL), and the other by the University of Bielefeld Center for Biotechnology (CeBiTec). After completion, the two annotations were subjected to a side-by-side comparison, in which discrepancies were examined and manually edited.

Annotation by ORNL proceeded similarly to [18] and is briefly described here. Genes were predicted using GLIMMER [104] and CRITICA [105]. The lists of predicted genes were merged with the start site from CRITICA being used when stop sites were identical. The predicted CDSs were translated and submitted to a BLAST analysis against the KEGG database [106]. The BLAST analysis was used to evaluate overlaps and alternative start sites. Genes with large overlaps where both had good (1e−40) BLAST hits were left for manual resolution. Remaining overlaps were resolved manually and a QA process was used to identify frameshifted, missing, and pseudogenes. The resulting list of predicted CDSs were translated, and these amino acid sequences were used to query the National Center for Biotechnology Information (NCBI) nonredundant database, UniProt, TIGRFam, Pfam, PRIAM, KEGG, COG, and InterPro databases. PFam and TIGRFam were run with scores > trusted cutoff scores for the hidden Markov models (HMMs). Product assignments were made based on the hierarchy of TIGRFam, PRIAM, Pfam, Smart (part of InterPro), UniProt, KEGG, and COG databases.

Annotation by CeBiTec began by calling genes using the REGANOR strategy [107], which is based on training GLIMMER [104] with a positive training set created by CRITICA [105]. Predicted CDSs were translated, and these amino acid sequences were used to query the NCBI nonredundant database, SwisProt, TIGRFam, Pfam, KEGG, COG, and InterPro databases. Results were collated and presented via GenDB (http://www.cebitec.uni-bielefeld.de/groups/brf/software/gendb_info/) [108] for manual verification. For each gene, the list of matches to databases was examined to deduce the gene product. Specific functional assignments suggested by matches with SwisProt and the NCBI nonredundant database were only accepted if they covered over 75% of the gene length, had an e-value < 0.001, and were supported by hits to curated databases (Pfam or TIGRFam, with scores greater than the trusted cutoff scores for the HMMs), or were consistent with gene context in the genome (e.g., membership in a potential operon with other genes with convincing matches to curated databases). When it was not possible to clarify the function of a gene based on matches in SwissProt and the nonredundant database, but evolutionary relatedness was apparent (e.g., membership in a Pfam with a score greater than the trusted cutoff score for the family HMM), genes were annotated as members of gene families.

When it was not possible to infer function or family membership, genes were annotated as encoding hypothetical or conserved hypothetical proteins. If at least three matches from three other species that covered >75% of the gene's length were retrieved from SwissProt and the nonredundant database, the genes were annotated as encoding conserved hypothetical proteins. Otherwise, the presence of a Shine-Dalgarno sequence upstream from the predicted start codon was verified and the gene was annotated as encoding a hypothetical protein. For genes encoding either hypothetical or conserved hypothetical proteins, the cellular location of their potential gene products was inferred based on TMHMM and SignalP [109,110]. When transmembrane alpha helices were predicted by TMHMM, the gene product was annotated as a predicted membrane protein. When SignalP Sigpep probability and max cleavage site probability were both >0.75, and no other predicted transmembrane regions were present, the gene was annotated as a predicted periplasmic or secreted protein.

### Comparative genomics.

All CDSs for this genome were used to query the TransportDB database [111]. Matches were assigned to transporter families to facilitate comparisons with other organisms within the TransportDB database (http://www.membranetransport.org/). To compare operon structure for genes encoding the Calvin-Benson-Bassham cycle, amino acid biosynthesis, phosphonate metabolism, and to find all of the genes encoding MCPs, BLAST-queries of the microbial genomes included in the Integrated Microbial Genomes database were conducted [112]. Comparison of operon structure was greatly facilitated by using the “Show Neighborhoods” function available on the IMG website (http://img.jgi.doe.gov/).

## Supporting Information

Figure S1 Phylogenetic Relationships of SoxC Sequences of *Thiom. crunogena* XCL-2 with SoxC/SorA Sequences of Selected Bacteria Sequences were aligned using the program package MacVector. Neighbor-joining and parsimony trees based on the predicted amino acid sequences were calculated using PAUP 4.0b10. At the base of the three main groups, bootstrap values (1,000 replicates) are given for the neighbor-joining (first value) and parsimony analyses (second value). Bootstrap values are depicted only at the base of the three main groups. Arabidopsis thaliana represents a plant assimilative nitrate reductase and Drosophila melanogaster represents a eukaryotic sulfite oxidase.
(759 KB TIF)

Figure S2 Phylogenetic Relationships of PhnJ Sequences of *Thiom. crunogena* with PhnJ Sequences of Selected Bacteria Sequences were aligned using the program package MacVector. Neighbor-joining and parsimony trees based on the predicted amino acid sequences were calculated using PAUP 4.0b10. Bootstrap values (1,000 replicates; neighbor-joining/parsimony analyses) are depicted only at the base of the three main groups and for the branch grouping *Thiob. denitrificans* and *Thiom. crunogena.*
(892 KB TIF)

Protocol S1 Nitrogen Uptake and Assimilation (49 KB DOC)

Table S1 Proteins with a Heme-Coordinating Motif (CxxCH) (49 KB DOC)

Table S2 Growth of *Thiom. crunogena* XCL-2 on Solid Artificial Seawater Medium Supplemented with Carbon and Electron Sources (31 KB DOC)

Table S3 *Thiom. crunogena* XCL-2 RubisCO Activity When Grown in the Presence and Absence of Organic Carbon (28 KB DOC)

Table S4 Percent Similarities and Identities of Proteobacterial *cbbL, cbbM,* and *cbbP* Genes (29 KB DOC)

Table S5 Amino Acid Biosynthesis Gene Organization in *Thiomi. crunogena* XCL-2 versus E. coli
(29 KB DOC)

Table S6 *Thiom. crunogena* XCL-2 Regulatory and Signaling Proteins (55 KB DOC)

### Accession Numbers

The GenBank (http://www.ncbi.nlm.nih.gov/Genbank) nonredundant database accession number for the complete sequence of the *Thiom. crunogena* XCL-2 genome is CP000109.

## Abbreviations

bp: base pairs
CAC: citric acid cycle
CDS: coding sequence
FI: form I
FII: form II
HMM: hidden Markov model
MCP: methyl-accepting chemotaxis protein

## References

[pbio-0040383-b001] Karl DM, Wirsen CO, Jannasch HW (1980). Deep-sea primary production at the Galápagos hydrothermal vents. Science.

[pbio-0040383-b002] Edwards KJ, Rogers DR, Wirsen CO, McCollom TM (2003). Isolation and characterization of novel psychrophilic, neutrophilic, Fe-oxidizing, chemolithoautotrophic alpha- and, gamma-Proteobacteria from the deep sea. Appl Environ Microbiol.

[pbio-0040383-b003] Kelley DS, Karson JA, Fruh-Green GL, Yoerger DR, Shank TM (2005). A serpentinite-hosted ecosystem: The lost city hydrothermal field. Science.

[pbio-0040383-b004] Johnson KS, Childress JJ, Beehler CL (1988). Short term temperature variability in the Rose Garden hydrothermal vent field. Deep-Sea Res.

[pbio-0040383-b005] Goffredi SK, Childress JJ, Desaulniers NT, Lee RW, Lallier FH (1997). Inorganic carbon acquisition by the hydrothermal vent tubeworm Riftia pachyptila depends upon high external P-CO2 and upon proton-equivalent ion transport by the worm. J Exp Biol.

[pbio-0040383-b006] Jannasch H, Wirsen C, Nelson D, Robertson L (1985). Thiomicrospira crunogena sp. nov., a colorless, sulfur-oxidizing bacterium from a deep-sea hydrothermal vent. Int J Syst Bacteriol.

[pbio-0040383-b007] Wirsen CO, Brinkhoff T, Kuever J, Muyzer G, Molyneaux S (1998). Comparison of a new *Thiomicrospira* strain from the Mid-Atlantic Ridge with known hydrothermal vent isolates. Appl Environ Microbiol.

[pbio-0040383-b008] Muyzer G, A, Teske C.O, Wirsen, Jannasch H.W (1995). Phylogenetic relationships of *Thiomicrospira* species and their identification in deep-sea hydrothermal vent samples by denaturing gradient gel electrophoresis of 16S rDNA fragments. Arch Microbiol.

[pbio-0040383-b009] Brinkhoff T, Sievert SM, Kuever J, Muyzer G (1999). Distribution and diversity of sulfur-oxidizing Thiomicrospira spp. at a shallow-water hydrothermal vent in the Aegean Sea (Milos, Greece). Appl Environ Microbiol.

[pbio-0040383-b010] Ruby EG, Wirsen CO, Jannasch HW (1981). Chemolithotrophic sulfur-oxidizing bacteria from the Galapagos Rift hydrothermal vents. Appl Environ Microbiol.

[pbio-0040383-b011] Ruby EG, Jannasch HW (1982). Physiological characteristics of Thiomicrospira sp. strain L-12 isolated from deep-sea hydrothermal vents. J Bacteriol.

[pbio-0040383-b012] Wirsen CO, Brinkhoff T, Kuever J, Muyzer G, Jannasch HW (1998). Comparison of a new *Thiomicrospira* strain from the Mid-Atlantic Ridge with known hydrothermal vent isolates. Appl Environ Microbiol.

[pbio-0040383-b013] Scott KM, Bright M, Fisher CR (1998). The burden of independence: Inorganic carbon utilization strategies of the sulphur chemoautotrophic hydrothermal vent isolate Thiomicrospira crunogena and the symbionts of hydrothermal vent and cold seep vestimentiferans. Cah Biol Mar.

[pbio-0040383-b014] Dobrinski KP, Longo DL, Scott KM (2005). A hydrothermal vent chemolithoautotroph with a carbon concentrating mechanism. J Bacteriol.

[pbio-0040383-b015] Ahmad A, Barry JP, Nelson DC (1999). Phylogenetic affinity of a wide, vacuolate, nitrate-accumulating Beggiatoa sp. from Monterey Canyon, California, with Thioploca spp. Appl Environ Microbiol.

[pbio-0040383-b016] Dufresne A, Salanoubat M, Partensky F, Artiguenave F, Axmann I (2003). Genome sequence of the cyanobacterium Prochlorococcus marinus SS120, a nearly minimal oxyphototrophic genome. Proc Natl Acad Sci U S A.

[pbio-0040383-b017] Palenik B, Brahamsha B, Larimer FW, Land M, Hauser L (2003). The genome of a motile marine *Synechococcus*. Nature.

[pbio-0040383-b018] Chain P, Lamerdin J, Larimer F, Regala W, Lao V (2003). Complete genome sequence of the ammonia-oxidizing bacterium and obligate chemolithoautotroph Nitrosomonas europaea. J Bacteriol.

[pbio-0040383-b019] Larimer F, Chain P, Hauser L, Lamerdin J, Malfatti S (2004). Complete genome sequence of the metabolically versatile photosynthetic bacterium Rhodopseudomonas palustris. Nature Biotechnol.

[pbio-0040383-b020] Eisen JA, Nelson KE, Paulsen IT, Heidelberg JF, Wu M (2002). The complete genome sequence of Chlorobium tepidum TLS, a photosynthetic, anaerobic, green-sulfur bacterium. Proc Natl Acad Sci U S A.

[pbio-0040383-b021] Beller HR, Chain PSG, Letain TE, Chakicherla A, Larimer FW (2006). The genome sequence of the obligately chemolithoautotrophic, facultatively anaerobic bacterium Thiobacillus denitrificans. J Bacteriol.

[pbio-0040383-b022] Deckert G, Warren PV, Gaasterland T, Young WG, Lenox AL (1998). The complete genome of the hyperthermophilic bacterium Aquifex aeolicus. Nature.

[pbio-0040383-b023] Lowe TM, Eddy SR (1997). tRNAscan-SE: A program for improved detection of transfer RNA genes in genomic sequence. Nucleic Acids Res.

[pbio-0040383-b024] Casjens S (2003). Prophages and bacterial genomics: What have we learned so far?. Mol Microbiol.

[pbio-0040383-b025] Lucchini S, Desiere F, Brussow H (1999). Similarly organized lysogeny modules in temperate Siphoviridae from low GC content gram positive bacteria. Virology.

[pbio-0040383-b026] Weigele PR, Sampson L, Winn-Stapley D, Casjens SR (2005). Molecular genetics of bacteriophage P22 scaffolding protein's functional domains. J Mol Biol.

[pbio-0040383-b027] Sanger F, Coulson AR, Hong GF, Hill DF, Petersen GB (1982). Nucleotide sequence of bacteriophage lambda DNA. J Mol Biol.

[pbio-0040383-b028] Rohwer F, Segall A, Steward G, Seguritan V, Breitbart M (2000). The complete genomic sequence of the marine phage Roseophage SIO1 shares homology with non-marine phages. Limnol Oceanogr.

[pbio-0040383-b029] Sternberg N, Weisberg R (1977). Packaging of coliphage lambda DNA. II. The role of gene D protein. J Mol Biol.

[pbio-0040383-b030] Paul JH, Sullivan MB (2005). Marine phage genomics: What have we learned?. Curr Opin Biotech.

[pbio-0040383-b031] Canchaya C, Proux C, Fournous G, Bruttin A, Brussow H (2003). Prophage genomics. Microbiol Mol Biol Rev.

[pbio-0040383-b032] Friedrich CG, Quentmeier A, Bardischewsky F, Rother D, Kraft R (2000). Novel genes coding for lithotrophic sulfur oxidation of Paracoccus pantotrophus GB17. J Bacteriol.

[pbio-0040383-b033] Rother D, Henrich HJ, Quentmeier A, Bardischewsky F, Friedrich CG (2001). Novel genes of the sox gene cluster, mutagenesis of the flavoprotein SoxF, and evidence for a general sulfur-oxidizing system in Paracoccus pantotrophus GB17. J Bacteriol.

[pbio-0040383-b034] Friedrich CG, Bardischewsky F, Rother D, Quentmeier A, Fischer J (2005). Prokaryotic sulfur oxidation. Curr Opin Microbiol.

[pbio-0040383-b035] Schutz M, Maldener I, Griesbeck C, Hauska G (1999). Sulfide-quinone reductase from *Rhodobacter capsulatus:* Requirement for growth, periplasmic localization, and extension of gene sequence analysis. J Bacteriol.

[pbio-0040383-b036] Javor BJ, Wilmot DB, Vetter RD (1990). pH–Dependent metabolism of thiosulfate and sulfur globules in the chemolithotrophic marine bacterium *Thiomicrospira crunogena*. Arch Microbiol.

[pbio-0040383-b037] Friedrich CG, Rother D, Bardischewsky F, Quentmeier A, Fischer J (2001). Oxidation of reduced inorganic sulfur compounds by bacteria: Emergence of a common mechanism?. Appl Environ Microbiol.

[pbio-0040383-b038] Fani R, Brilli M, Lio P (2005). The origin and evolution of operons: The piecewise building of the proteobacterial histidine operon. J Mol Evol.

[pbio-0040383-b039] Price MN, Huang KH, Arkin AP, Alm EJ (2005). Operon formation is driven by co-regulation and not by horizontal gene transfer. Genome Res.

[pbio-0040383-b040] Petri R, Podgorsek L, Imhoff JF (2001). Phylogeny and distribution of the soxB gene among thiosulfate-oxidizing bacteria. Fems Microbiol Lett.

[pbio-0040383-b041] Kelly DP, Shergill JK, Lu WP, Wood AP (1997). Oxidative metabolism of inorganic sulfur compounds by bacteria. Antonie Van Leeuwenhoek Intl J Gen Mol Microbiol.

[pbio-0040383-b042] Nelson DC, Hagen KD (1995). Physiology and biochemistry of symbiotic and free-living chemoautotrophic sulfur bacteria. Am Zool.

[pbio-0040383-b043] Schwartz E, Friedrich B, Dworkin M (2005). The H2-metabolizing prokaryotes. The prokaryotes: An evolving electronic resource for the microbiological community, release 3.14.

[pbio-0040383-b044] Nishihara H, Yaguchi T, Chung SY, Suzuki K, Yanagi M (1998). Phylogenetic position of an obligately chemoautotrophic, marine hydrogen-oxidizing bacterium, *Hydrogenovibrio marinus,* on the basis of 16S rRNA gene sequences and two form I RubisCO gene sequences. Arch Microbiol.

[pbio-0040383-b045] Nishihara H, Miyata Y, Miyashita Y, Bernhard M, Pohlmann A (2001). Analysis of the molecular species of hydrogenase in the cells of an obligately chemolithoautotrophic, marine hydrogen-oxidizing bacterium, Hydrogenovibrio marinus. Biosci Biotechnol Biochem.

[pbio-0040383-b046] Nishihara H, Igarashi Y, Kodama T (1991). Hydrogenovibrio marinus gen. nov., sp. nov., a marine obligately chemolithoautotrophic hydrogen-oxidizing bacterium. Int J Syst Bacteriol.

[pbio-0040383-b047] Dross F, Geisler V, Lenger R, Theis F, Krafft T (1992). The quinone-reactive Ni/Fe-hydrogenase of Wollinella succinogenes. Eur J Biochem.

[pbio-0040383-b048] Friedrich T, Scheide D (2000). The respiratory complex I of bacteria, archaea and eukarya and its module common with membrane-bound multisubunit hydrogenases. FEBS Letters.

[pbio-0040383-b049] Smith M, Finel M, Korolik V, Mendz G (2000). Characteristics of the aerobic respiratory chains of the microaerophiles Campylobacter jejuni and Helicobacter pylori. Arch Microbiol.

[pbio-0040383-b050] Steuber J (2001). Na+ translocation by bacterial NADH:quinone oxidoreductases: An extension to the complex-I family of primary redox pumps. Biochim Biophys Acta.

[pbio-0040383-b051] Kumagai H, Fujiwara T, Matsubara H, Saeki K (1997). Membrane localization, topology, and mutual stabilization of the rnfABC gene products in Rhodobacter capsulatus and implications for a new family of energy-coupling NADH oxidoreductases. Biochemistry.

[pbio-0040383-b052] Lange BM, Rujan T, Martin W, Croteau R (2000). Isoprenoid biosynthesis: The evolution of two ancient and distinct pathways across genomes. Proc Natl Acad Sci U S A.

[pbio-0040383-b053] Hedl M, Sutherlin A, Wilding E, Mazzulla M, McDevitt D (2002). Enterococcus faecalis acetoacetyl-coenzyme A thiolase/3-hydroxy-3-methylglutaryl-coenzyme A reductase, a dual-function protein of isopentenyl diphosphate biosynthesis. J Bacteriol.

[pbio-0040383-b054] Wilding EI, Brown JR, Bryant AP, Chalker AF, Holmes DJ (2000). Identification, evolution, and essentiality of the mevalonate pathway for isopentenyl diphosphate biosynthesis in Gram-positive cocci. J Bacteriol.

[pbio-0040383-b055] Humbelin M, Thomas A, Lin J, Li J, Jore J (2002). Genetics of isoprenoid biosynthesis in Paracoccus zeaxanthinifaciens. Gene.

[pbio-0040383-b056] Preisig O, Zufferey R, Thony-Meyer L, Appleby C, Hennecke H (1996). A high-affinity cbb3-type cytochrome oxidase terminates the symbiosis-specific respiratory chain of Bradyrhizobium japonicum. J Bacteriol.

[pbio-0040383-b057] Hooper AB, Arciero DM, Bergmann D, Hendrich MP, Zannoni D (2004). The oxidation of ammonia as an energy source in bacteria. Respiration in Archaea and Bacteria.

[pbio-0040383-b058] Jackson JB (2003). Proton translocation by transhydrogenase. FEBS Letts.

[pbio-0040383-b059] Boonstra B, French CE, Wainwright I, Bruce NC (1999). The udhA gene of Escherichia coli encodes a soluble pyridine nucleotide transhydrogenase. J Bacteriol.

[pbio-0040383-b060] Mori S, Kawai S, Shi F, Mikami B, Murata K (2005). Molecular conversion of NAD kinase to NADH kinase through single amino acid residue substitution. J Biol Chem.

[pbio-0040383-b061] Paulsen IT, Nguyen L, Sliwinski MK, Rabus R, Saier MH (2000). Microbial genome analyses: Comparative transport capabilities in eighteen prokaryotes. J Mol Biol.

[pbio-0040383-b062] Ren Q, Paulsen IT (2005). Comparative analyses of fundamental differences in membrane transport capabilities in prokaryotes and eukaryotes. PLoS Comput Biol.

[pbio-0040383-b063] Badger MR, Price GD, Long BM, Woodger FJ (2006). The environmental plasticity and ecological genomics of the cyanobacterial CO2 concentrating mechanism. J Exp Bot.

[pbio-0040383-b064] Smith KS, Ferry JG (2000). Prokaryotic carbonic anhydrases. FEMS Microbiol Rev.

[pbio-0040383-b065] So AK, Espie GS, Williams EB, Shively JM, Heinhorst S (2004). A novel evolutionary lineage of carbonic anhydrase (epsilon class) is a component of the carboxysome shell. J Bacteriol.

[pbio-0040383-b066] Sawaya MR, Cannon GC, Heinhorst S, Tanaka S, Williams EB (2006). The structure of beta-carbonic anhydrase from the carboxysomal shell reveals a distinct subclass with one active site for the price of two. J Biol Chem.

[pbio-0040383-b067] Felce J, Saier MH (2004). Carbonic anhydrase fused to anion transporters of the SulP family: Evidence for a novel type of bicarbonate transporter. J Mol Microbiol Biotechnol.

[pbio-0040383-b068] Yoshizawa Y, Toyoda K, Arai H, Ishii M, Igarashi Y (2004). CO2-responsive expression and gene organization of three ribulose-1,5-bisphosphate carboxylase/oxygenase enzymes and carboxysomes in Hydrogenovibrio marinus strain MH-110. J Bacteriol.

[pbio-0040383-b069] Hayashi NR, Arai H, Kodama T, Igarashi Y (1997). The novel genes, cbbQ and cbbO, located downstream from the RubisCO genes of *Pseudomonas hydrogenothermophila,* affect the conformational states and activity of RubisCO. Biochem Biophys Res Commun.

[pbio-0040383-b070] Hayashi NR, Arai H, Kodama T, Igarashi Y (1999). The cbbQ genes located downstream of the form I and form II RubisCO genes, affect the activity of both RubisCOs. Biochem Biophys Res Commun.

[pbio-0040383-b071] Badger M, Hanson D, Price GD (2002). Evolution and diversity of CO2 concentrating mechanisms in cyanobacteria. Funct Plant Biol.

[pbio-0040383-b072] Gibson JL, Tabita FR (1996). The molecular regulation of the reductive pentose phosphate pathway in Proteobacteria and Cyanobacteria. Arch Microbiol.

[pbio-0040383-b073] Kusian B, Bowien B (1997). Organization and regulation of cbb CO2 assimilation genes in autotrophic bacteria. FEMS Microbiol Rev.

[pbio-0040383-b074] Shively JM, Van Keulen G, Meijer WG (1998). Something from almost nothing: Carbon dioxide fixation in chemoautotrophs. Ann Rev Microbiol.

[pbio-0040383-b075] Falcone DL, Tabita FR (1993). Complementation analysis and regulation of CO2 fixation gene expression in a ribulose 1,5-bisphosphate carboxylase-oxygenase deletion strain of Rhodospirillum rubrum. J Bacteriol.

[pbio-0040383-b076] Ding YR, Ronimus RS, Morgan HW (2000). Sequencing, cloning, and high-level expression of the pfp gene, encoding a ppi-dependent phosphofructokinase from the extremely thermophilic eubacterium Dictyoglomus thermophilum. J Bacteriol.

[pbio-0040383-b077] Ronimus RS, Morgan HW (2001). The biochemical properties and phylogenies of phosphofructokinases from extremophiles. Extremophiles.

[pbio-0040383-b078] Wood AP, Aurikko JP, Kelly DP (2004). A challenge for 21st century molecular biology and biochemistry: What are the causes of obligate autotrophy and methanotrophy?. FEMS Microbiol Rev.

[pbio-0040383-b079] Hughes NJ, Clayton C, Chalk P, Kelly D (1998). Helicobacter pylori porCDAB and oorDABC genes encode distinct pyruvate:flavodoxin and 2-oxoglutarate: acceptor oxidoreductases which mediate electron transport to NADP. J Bacteriol.

[pbio-0040383-b080] Kather B, Stingl K, Van der Rest M, Altendorf K, Molenaar D (2000). Another unusual type of citric acid cycle enzyme in *Helicobacter pylori:* The malate:quinone oxidoreductase. J Bacteriol.

[pbio-0040383-b081] Gehring U, Arnon DI (1972). Purification and properties of alpha-ketoglutarate synthase from a photosynthetic bacterium. J Biol Chem.

[pbio-0040383-b082] Breese K, Boll M, Alt-Morbe J, Schagger H, Fuchs G (1998). Genes coding for the benzoyl-CoA pathway of anaerobic aromatic metabolism in the bacterium Thauera aromatica. Eur J Biochem.

[pbio-0040383-b083] Jahn D, Verkamp E, Soll D (1992). Glutamyl-transfer RNA: A precursor of heme and chlorophyll biosynthesis. Trends Biochem Sci.

[pbio-0040383-b084] van Veen HW (1997). Phosphate transport in prokaryotes: Molecules, mediators and mechanisms. Antonie Van Leeuwenhoek Intl J Gen Molec Microbiol.

[pbio-0040383-b085] Prere MF, Chandler M, Fayet O (1990). Transposition in *Shigella dysenteriae:* isolation and analysis of IS911, a new member of the IS3 group of insertion sequences. J Bacteriol.

[pbio-0040383-b086] Dyhrman ST, Chappell PD, Haley ST, Moffett JW, Orchard ED (2006). Phosphonate utilization by the globally important marine diazotroph *Trichodesmium*. Nature.

[pbio-0040383-b087] Kolowith LC, Ingall ED, Benner R (2001). Composition and cycling of marine organic phosphorus. Limnol Oceanogr.

[pbio-0040383-b088] Klotz MG, Arp DJ, Chain PSG, El-Sheikh AF, Hauser LJ (2006). The complete genome sequence of the marine, chemolithoautotrophic, ammonia-oxidizing bacterium Nitrosococcus oceani ATCC19707. Appl Environ Microbiol.

[pbio-0040383-b089] Watson SW (1965). Characteristics of a marine nitrifying bacterium, Nitrosocystis oceanus Sp. N. Limnol Oceanogr.

[pbio-0040383-b090] Romling U, Gomelsky M, Galperin MY (2005). C-di-GMP: The dawning of a novel bacterial signalling system. Molec Microbiol.

[pbio-0040383-b091] Zhulin I, Taylor B, Dixon R (1997). PAS domain S-boxes in Archaea, Bacteria and sensors for oxygen and redox. Trends Biochem Sci.

[pbio-0040383-b092] McCarter LL (2001). Polar flagellar motility of the Vibrionaceae. Microbiol Mol Biol Rev.

[pbio-0040383-b093] Wadhams GH, Armitage JP (2004). Making sense of it all: Bacterial chemotaxis. Nature Rev Molec Cell Biol.

[pbio-0040383-b094] Kachlany SC, Planet PJ, DeSalle R, Fine DH, Figurski DH (2001). Genes for tight adherence of Actinobacillus actinomycetemcomitans: From plaque to plague to pond scum. Trends Microbiol.

[pbio-0040383-b095] Jannasch HW, Mottl MJ (1985). Geomicrobiology of deep-sea hydrothermal vents. Science.

[pbio-0040383-b096] McCollom TM, Shock EL (1997). Geochemical constraints on chemolithoautotrophic metabolism by microorganisms in seafloor hydrothermal systems. Geochim Cosmochim Acta.

[pbio-0040383-b097] Nies DH (2003). Efflux-mediated heavy metal resistance in prokaryotes. FEMS Microbiol Rev.

[pbio-0040383-b098] Hou S, Saw JH, Lee KS, Freitas TA, Belisle C (2004). Genome sequence of the deep-sea gamma-proteobacterium Idiomarina loihiensis reveals amino acid fermentation as a source of carbon and energy. Proc Natl Acad Sci U S A.

[pbio-0040383-b099] Nies DH (1999). Microbial heavy-metal resistance. Appl Microbiol Biotechnol.

[pbio-0040383-b100] Edgcomb VP, Molyneaux SJ, Saito MA, Lloyd K, Boer S (2004). Sulfide ameliorates metal toxicity for deep-sea hydrothermal vent archaea. Appl Environ Microbiol.

[pbio-0040383-b101] Ewing BL, Hillier M, Wendl P, Green P (1998). Basecalling of automated sequencer traces using phred. I. Accuracy assessment. Genome Res.

[pbio-0040383-b102] Ewing B, Green P (1998). Basecalling of automated sequencer traces using phred. II. Error probabilities. Genome Res.

[pbio-0040383-b103] Gordon D, Abajian C, Green P (1998). Consed: A graphical tool for sequence finishing. Genome Res.

[pbio-0040383-b104] Delcher AL, Harmon D, Kasif S, White O, Salzberg SL (1999). Improved microbial gene identification with GLIMMER. Nucleic Acids Res.

[pbio-0040383-b105] Badger JH, Olsen GJ (1999). CRITICA: Coding region identification tool invoking comparative analysis. Molec Biol Evol.

[pbio-0040383-b106] Kanehisa M, Goto S (2000). KEGG: Kyoto Encyclopedia of Genes and Genomes. Nucleic Acids Res.

[pbio-0040383-b107] McHardy AC, Goesmann A, Puhler A, Meyer F (2004). Development of joint application strategies for two microbial gene finders. Bioinformatics.

[pbio-0040383-b108] Meyer F, Goesmann A, McHardy AC, Bartels D, Bekel T (2003). GenDB—An open source genome annotation system for prokaryotic genomes. Nucleic Acids Res.

[pbio-0040383-b109] Krogh A, Larsson B, von Heijne G, Sonnhammer ELL (2001). Predicting transmembrane protein topology with a hidden Markov model: Application to complete genomes. J Molec Biol.

[pbio-0040383-b110] Bendtsen JD, Nielsen H, von Heijne G, Brunak S (2004). Improved prediction of signal peptides: SignalP 3.0. J Molec Biol.

[pbio-0040383-b111] Ren Q, Kang KH, Paulsen IT (2004). TransportDB: A relational database of cellular membrane transport systems. Nucleic Acids Res.

[pbio-0040383-b112] Markowitz VM, Korzeniewski F, Palaniappan K, Szeto E, Werner G (2006). The integrated microbial genomes (IMG) system. Nucl Acids Res.

[pbio-0040383-b113] Swofford DL (2002). PAUP*. Phylogenetic analysis using parsimony (*and other methods), version 4. [computer program].

[pbio-0040383-b114] Thompson JD, Gibson TJ, Plewniak F, Jeanmougin F, Higgins DG (1997). The CLUSTAL X windows interface: Flexible strategies for multiple sequence alignment aided by quality analysis tools. Nucl Acids Res.

