# Supplementary material for: The Genome of Deep-Sea Vent Chemolithoautotroph Thiomicrospira crunogena XCL-2
Source: PLoS Biol. 2006 Nov 14;4(12):e383. doi: 10.1371/journal.pbio.0040383 (PMC1635747; doi:10.1371/journal.pbio.0040383)
Supplement: Supplementary file 1 [file pbio.0040383.sd001.doc]

**Supporting protocol S1.** Nitrogen uptake and assimilation

*Thiomicrospira crunogena* XCL-2 is capable of growing with nitrate or ammonia as its nitrogen source ([6]; K. Scott, unpubl. data). Accordingly, it has an apparent operon encoding the components of a NasFED-type nitrate transporter (*Tcr1153 - 5*) [115], cytoplasmic assimilatory nitrate (*nasA; Tcr1159)* and nitrite reductase (*nirBD; Tcr1157-8)* genes, as well as four Amt-family ammonia transporters (*Tcr0954; Tcr1340; Tcr1500; Tcr2151*).

Ammonia originating from environmental sources or produced from nitrate reduction is incorporated into the *T. crunogena* XCL-2 organic nitrogen pool by glutamine synthetase and NADPH-dependent glutamate synthase. *T. crunogena* XCL-2 has three different glutamine synthetase genes: one encodes a GlnA-type enzyme (*Tcr0536*) while the others are both GlnT-type (*Tcr1347, Tcr1798*) [116]. Perhaps **these three glutamine synthetase genes are differentially expressed under different nitrogen conditions.**

Genes encoding the majority of the enzymes necessary to synthesize all 20 L-amino acids were detected; exceptions and omissions are described here. The first enzyme of aromatic amino acid synthesis, 3-deoxy-D-arabino-heptulosonate 7-phosphate (DAHP) synthetase, is encoded by a single gene. More commonly, bacteria have 2-3 isoforms of this enzyme, which are differentially regulated by the concentrations of tyrosine, phenylalanine, and tryptophan [117]. *T. crunogena* XCL-2 also has a single copy of the genes encoding the large and small subunits of acetolactate synthase, which is the “gatekeeper” enzyme of valine, leucine, and isoleucine biosynthesis, while *E. coli* has three isoforms that are differentially sensitive to leucine, valine and isoleucine [118]. It would be of interest to determine whether the single isoforms of these enzymes present in *T. crunogena* XCL-2 are sensitive to the concentration of any of their three amino acid endproducts.

Only a single gene was identified for chorismate mutase, as part of a PheA-like bifunctional protein for the production of phenylpyruvate for phenylalanine synthesis. How the prephenate necessary for tyrosine synthesis is supplied is not apparent. The gene encoding 4-hydroxyphenylpyruvate-producing prephenate dehydrogenase (*tyrC)* does not appear to be bifunctional, as it lacks a domain that could catalyze the chorismate mutase step. Searches for *aroQ* and *aroH*-type chorismate mutase genes [119] yielded only *pheA.*

With regard to lysine synthesis, the identity of the gene encoding N-succinyl-LL-diaminopimelate aminotransferase is not clear. Currently, the gene (*dapC*) encoding this enzyme has only been unambiguously identified in *Bordetella pertussis* [120]. A BLAST search of the *T. crunogena* XCL-2 genome with the *B. pertussis* DapC amino acid sequence did not yield an apparent homolog. A gene identified as *argD* is present, which encodes N-acetyl-ornithine aminotransferase, which can also catalyze the DapC reaction [121]. However, it is unclear whether the *argD* gene product functions in lysine synthesis, as cell extracts from other species contain a heretofore unidentified enzyme specific to N-succinyl-LL-diaminopimelate whose gene locus has not been determined [121].

Alanine synthesis is perplexing in this organism, as genes encoding alanine dehydrogenase, alanine-oxo-acid transaminase, alanine transaminase and alanine-glyoxylate transaminase are all absent, nor are the enzymes present necessary to synthesize alanine from cysteine. Perhaps one of the many transaminase genes detected, the specificity of which could not be deduced based on its sequence, is capable of using pyruvate as a substrate.

Amino acid degradation capabilities are quite limited, as in the obligate chemoautotroph *Nitrosomonas europaea* [18]. The genome sequence was searched for amino acid-catabolizing enzymes common in other bacteria, and many were lacking. For example, both histidine and serine ammonia-lyase are absent. Enzymes necessary for histidine degradation via urocanate or histamine are all absent. Aromatic amino acids cannot be degraded, based on the lack of genes encoding tryptophanase, tryptophan monoxygenase, aromatic-L-amino-acid decarboyxlase, aromatic-amino-acid transaminase, tyrosine aminotransferase, or any of the other enzymes necessary to recycle their carbon skeletons through central carbon metabolism. Carbon skeletons from valine, isoleucine, and leucine cannot be routed to central carbon metabolism via acetyl-CoA or succinyl-CoA, as acyl-CoA dehydrogenase and enoyl-CoA hydratase genes are not present.

With the exceptions of glutamine and asparagine, aminoacyl-tRNA synthetases for all amino acids are present. Two copies of the *gltX* gene are present, and it is not possible to deduce whether they have differential specificities for glutamine or glutamate based on their sequences. In *T. crunogena* XCL-2*,* as in other organisms [122], GltX is probably capable of aminoacylating both tRNA(glu) and tRNA(gln) with glutamate, as glutaminyl-tRNA synthetase is missing. Genes for the three subunits of a tRNA-dependent amidotransferase (adt) are present, and their gene products could convert the glutamyl-tRNA(glu) and aspartyl-tRNA(asp) to glutaminyl-tRNA(gln) and aspartanyl-tRNA(asn). Glycyl- and phenylalanyl-tRNA synthetases are present as heterodimeric forms. In addition to a canonical histidinyl-tRNA synthetase gene, a paralog is present (*hisZ*) and may function as a regulatory protein that interacts with ATP phosphoribosyltransferase, which catalyzes the first step of histidine synthesis [123]. The paralog may act as an intracellular histidine sensor [123]. Two copies of lysyl-tRNA synthetase genes are also present. One is truncated (as in [124]), and may have a different function.

The arginine, tryptophan, leucine, and histidine operons present in the Enterobacteria, Pasteurellales, Vibrionales, and Alteromonidales are disrupted in *T. crunogena* (Table S1), as they are in other “deeply branching” members of the γ-Proteobacteria (*Nitrosococcus oceani, Methylococcus capsulatus* Bath, and *Pseudomonas aeruginosa;* K. Scott, unpubl. data).

Nucleotide metabolism

Genes encoding all of the enzymes necessary to synthesize purine and pyrimidine nucleotides are present. However, the salvage enzymes are poorly represented, as in *Nitrosomonas europaea* [18]. Adenosine and cytidine deaminases are absent, as are purine or pyrimidine phosphorylases and hydrolases. Since *T. crunogena* XCL-2 is an obligate autotroph, an extensive arsenal of salvage enzymes may not be necessary, as it probably does not rely on environmental sources of these compounds either for nucleotide synthesis, or to use as carbon sources.

**References**

115. Lin JT, Goldman BS, Stewart V (1994) The Nasfedcba Operon for Nitrate and Nitrite Assimilation in *Klebsiella pneumoniae* M5al. J Bacteriol 176: 2551-2559.

116. Merrick MJ, Edwards RA (1995) Nitrogen control in bacteria. Microbiol Rev 59: 604-622.

117. Jensen RA, Nasser DS, Nester EW (1967) Comparative control of a branch-point enzyme in microorganisms. J Bacteriol 94: 1582-1593.

118. Lawther RP, Wek RC, Lopes JM, Pereira R, Taillon BE, et al. (1987) The complete nucleotide sequence of the *ilvGMEDA* operon of *Escherichia coli* K-12. Nucleic Acids Res 15: 2137-2155.

119. Calhoun D, Bonner C, Gu W, Xie G, Jensen R (2001) The emerging periplasm-localized subclass of AroQ chorismate mutases, exemplified by those from *Salmonella typhimurium* and *Pseudomonas aeruginosa*. Genome Biol 2: research0030.0031 - research0030.0016.

120. Fuchs TM, Schneider B, Krumbach K, Eggeling L, Gross R (2000) Characterization of a *Bordetella pertussis* Diaminopimelate (DAP) Biosynthesis Locus Identifies dapC, a Novel Gene Coding for an N-Succinyl-L,L-DAP Aminotransferase. J Bacteriol 182: 3626-3631.

121. Cox RJ, Wang PSH (2001) Is N-acetylornithine aminotransferase the real N-succinyl-LL-diaminopimelate aminotransferase in *Escherichia coli* and *Mycobacterium smegmatis*? J Chem Soc-Perkin Transactions 1: 2006-2008.

122. Akochy PM, Bernard D, Roy PH, Lapointe J (2004) Direct glutaminyl-tRNA biosynthesis and indirect asparaginyl-tRNA biosynthesis in *Pseudomonas aeruginosa* PAO1. J Bacteriol 186: 767-776.

123. Champagne KS, Sissler M, Larrabee Y, Doublie S, Francklyn CS (2005) Activation of the Hetero-octameric ATP Phosphoribosyl Transferase through Subunit Interface Rearrangement by a tRNA Synthetase Paralog. J Biol Chem 280: 34096-34104.

124. Kong L, Fromant M, Blanquet S, Plateau P (1991) Evidence for a new *Escherichia coli* protein resembling a lysyl-tRNA synthetase. Gene 108: 163-164.

125. Scott KM, Schwedock J, Schrag DP, Cavanaugh CM (2004) Influence of form IA RubisCO and environmental dissolved inorganic carbon on the δ^13^C of the clam-bacterial chemoautotrophic symbiosis *Solemya velum*. Environ Microbiol 6: 1210-1219.
