# Supplementary material for: The Genome of Deep-Sea Vent Chemolithoautotroph Thiomicrospira crunogena XCL-2
Source: PLoS Biol. 2006 Nov 14;4(12):e383. doi: 10.1371/journal.pbio.0040383 (PMC1635747; doi:10.1371/journal.pbio.0040383)
Supplement: Supplementary file 2 [file pbio.0040383.st001.doc]

**TABLE S1.** Proteins with a heme-coordinating motif (CxxCH)

| Predicted location | Locus tag # | Predicted function |
| --- | --- | --- |
| Cytoplasm | *Tcr0028* | Cyclic diguanylate phosphodiesterase |
|  | *Tcr0157* | SoxD |
|  | *Tcr0324* | UvrABC system protein A |
|  | *Tcr0387* | ThiF family protein |
|  | *Tcr0719* | DNA-directed DNA polymerase |
|  | *Tcr0871* | Chaperone protein DnaJ |
|  | *Tcr0994* | DNA repair protein RadA |
|  | *Tcr1506* | Ribonuclease, Rne/Rng family protein |
|  | *Tcr1803* | Beta-lactamase family protein |
|  | *Tcr1975* | Conserved hypothetical protein |
| Membrane | *Tcr0993* | *bc_1_* complex cytochrome *c_1_* subunit |
|  | *Tcr1963* | *cbb_3_* cytochrome c oxidase CcoP subunit |
|  | *Tcr1964* | *cbb_3_* cytochrome c oxidase CcoO subunit |
|  | *Tcr0776* | Diguanylate cyclase/phosphodiesterase |
|  | *Tcr1187* | Hypothetical protein |
| Periplasm | *Tcr0601* | SoxA |
|  | *Tcr0604* | SoxX |
|  | *Tcr0111* | Thioredoxin-related protein |
|  | *Tcr0865* | Di-heme hypothetical protein |
|  | *Tcr1063* | Penta-heme hypothetical protein |
|  | *Tcr2105* | COG3258 single domain family |
|  | *Tcr1575* | COG2010 Ccca protein family; Cu detox.? |
|  | *Tcr2114* | *“* |
|  | *Tcr1266* | Hypothetical protein; similar to *Daro3004* and *Tbd1840* |
|  | *Tcr1885* | Hypothetical small mono-heme cytochrome protein |
|  | *Tcr0952* | COG2863 family of cytochromes c553 |
|  | *Tcr0628* | “ and likely to transfer electrons to the *bc_1_* complex and *cbb_3_* cytochrome c oxidase |
|  | *Tcr0629* | “ |
|  |  |  |
