# Supplementary material for: The Genome of Deep-Sea Vent Chemolithoautotroph Thiomicrospira crunogena XCL-2
Source: PLoS Biol. 2006 Nov 14;4(12):e383. doi: 10.1371/journal.pbio.0040383 (PMC1635747; doi:10.1371/journal.pbio.0040383)
Supplement: Supplementary file 3 [file pbio.0040383.st002.doc]

**TABLE S2.** Growth of *Thiomicrospira crunogena* XCL-2 on solid artificial seawater medium supplemented with carbon and electron sources^a^

| **Supplement** | **Air (0.03% CO_2_) headspace** | **CO_2_-free air headspace** |
| --- | --- | --- |
| None | -^b^ | - |
| Glucose | - | - |
| Acetate | - | - |
| Yeast extract | - | - |
| Thiosulfate | + | - |
| Thiosulfate + glucose | + | - |
| Thiosulfate + acetate | + | - |
| Thiosulfate + yeast extract | + | - |

^a^Cells were cultivated in chemostats under dissolved inorganic carbon limitation in artificial seawater medium supplemented with thiosulfate [14], and were spread on solid artificial seawater media supplemented with thiosulfate and organic carbon sources (40 mM thiosulfate, 0.02% w/v acetate, 0.02% w/v glucose, or 0.1% w/v yeast extract). Two replicates of each plate were made: one set was incubated with an air headspace while the other set was incubated in a BBL Gaspak jar continuously purged with air that had passed through a 30 cm column of soda lime to remove atmospheric CO_2_.

^b^Symbols indicate the presence (+) and absence (-) of growth twelve days after inoculation.
