# Supplementary material for: The Genome of Deep-Sea Vent Chemolithoautotroph Thiomicrospira crunogena XCL-2
Source: PLoS Biol. 2006 Nov 14;4(12):e383. doi: 10.1371/journal.pbio.0040383 (PMC1635747; doi:10.1371/journal.pbio.0040383)
Supplement: Supplementary file 5 [file pbio.0040383.st004.doc]

**TABLE S4.** *Thiomicrospira crunogena* XCL-2 Rubisco activity when grown in the presence and absence of organic carbon ^a^

| **Supplement** | **Rubisco activity  (μmol min^-1^ mg protein^-1^)** |
| --- | --- |
| None | 0.41 ± 0.02 |
| Glucose | 0.32 ± 0.04 |
| Acetate | 0.36 ± 0.05 |
| Yeast extract | 0.42 ± 0.05 |

^a^Cells were grown in chemostats in artificial seawater supplemented with thiosulfate (40 mM) under dissolved inorganic carbon limitation [14], in the presence and absence of organic carbon (0.02% w/v acetate, 0.02% w/v glucose, or 0.1% w/v yeast extract). Cells were harvested (10,000g, 10 min, 4°C), sonicated (three 15-sec sonications in the presence of glass sonication beads), and assayed for protein [14] and ribulose 1,5-bisphosphate carboxylase/oxygenase (RubisCO) activity [125].
