# Supplementary material for: The Genome of Deep-Sea Vent Chemolithoautotroph Thiomicrospira crunogena XCL-2
Source: PLoS Biol. 2006 Nov 14;4(12):e383. doi: 10.1371/journal.pbio.0040383 (PMC1635747; doi:10.1371/journal.pbio.0040383)
Supplement: Supplementary file 6 [file pbio.0040383.st005.doc]

**TABLE S5.** Amino acid biosynthesis gene organization in *Thiomicrospira crunogena* XCL-2 vs. *Escherichia coli*

| Amino acid | *E. coli* gene clusters | *T. crunogena*  gene clusters |
| --- | --- | --- |
| Arginine | *argECBH*^a^ | Genes present individually |
| Tryptophan | *trpE(GD)(CF)BA*^b^ | *trpEGDC*  *trpFBA* |
| Threonine | *thrABC* | *thrAC*  *thrB* |
| Leucine | *leuABCD* | *leuA*  *leuCDB* |
| Ile, leu, val | *ilvEDA* | Genes present individually |
| Histidine | *hisGDC(NB)HAF(IE)*^c^ | *hisGD*  *hisC*  *hisN*  *hisBHAFE*  *hisI* |

^a^*argE* is divergently transcribed from GBH

^b^*trp(GD)* and *trp(CF)* are gene fusions of *trpG* and *trpD*, and *trpC* and *trpF*, respectively. ^c^*his(NB)* and *his(IE)* are gene fusions of *hisN* and *hisB* and *hisI* and *hisE.*
