# Supplementary material for: The Genome of Deep-Sea Vent Chemolithoautotroph Thiomicrospira crunogena XCL-2
Source: PLoS Biol. 2006 Nov 14;4(12):e383. doi: 10.1371/journal.pbio.0040383 (PMC1635747; doi:10.1371/journal.pbio.0040383)
Supplement: Supplementary file 7 [file pbio.0040383.st006.doc]

**TABLE S6.** *Thiomicrospira crunogena* XCL-2 regulatory and signaling proteins

| Number | Item | Locus Tag Number (Tcr…)^a^ | | |
| --- | --- | --- | --- | --- |
| 72 | Transcription/Elongation/Termination Factors | |  | |
|  |  | |  | |
| 6 | Sigma Factors | | 0747 1205 1771 1806 2134 2156 | |
| 6 | Anti/Anti-Anti Sigma Factors | | 0730 0972 1038 1390 1225 1478 | |
| 6 | Termination/Antitermination Factors | | 0126 0283 0769 1122 1151 1396 | |
| 2 | Elongation Factors | | 0515 0880 | |
| 52 | Transcription factors | | 0004 0035 0054 0188 0205 0425 0426 0440 0460 0470 0540 0541 0617 0786 0788 0852 0858 0868 0904 0906 0969 1057 1203 1224 1234 1301 1338 1382 1405 1418 1444 1446 1487 1555 1588 1660 1702 1782 1789 1862 1872 1877 1884 1928 1947 2002 2078 2101 2126 2183 2185 2187 | |
|  |  | |  | |
| 128 | Signal Transduction proteins | |  | |
|  |  | |  | |
|  | Chemotaxis Signal Transduction proteins (27 total) | |  | |
| 14 | Methyl-accepting chemotaxis proteins | | 0027 0077 0361 0553 0570 0574 0759 0777 1394 1436 1897 1984 1999 2004 | |
| 2 | CheA signal transduction histidine kinase | | 0750 1612 | |
| 3 | CheW protein | | 0728 0751 0755 | |
| 2 | response regulator receiver modulated CheW protein | | 1271 1476 | |
| 1 | MCP methyltransferase, CheR-type | | 0757 | |
| 2 | CheY, response regulator receiver | | 0748 0754 | |
| 1 | response regulator receiver modulated CheB methylesterase | | 0758 | |
| 1 | CheD, stimulates methylation of MCP proteins | | 1613 | |
| 1 | CheZ chemotaxis phosphatase | | 0749 | |
|  |  | |  | |
|  | Non-Chemotaxis Signal Transduction (102 total) | |  | |
| 17 | Signal Transduction Histidine Kinase | | 0005 0066 0189 0226 0542 0905 0968 1040 1041 1202 1383 1445 1913 1924 2068 2180 2189 |  |
| 5 | diguanylate phosphodiesterase | | 0028 0122 0770 0860 1888 |  |
| 1 | response regulator receiver modulated diguanylate phosphodiesterase | | 0227 |  |
| 23 | diguanylate cyclase | | 0021 0090 0109 0116 0197 0200 0379 0380 0397 0457 0500 0785 0861 1021 1062 1108 1143 1377 1404 1580 1851 1920 1937 |  |
| 16 | diguanylate cyclase/phosphodiesterase | | 0152 0208 0776 1080 1089 1150 1222 1297 1773 1820 1931 2008 2024 2048 2190 2131 |  |
| 1 | response regulator receiver modulated diguanylate cyclase/phosphodiesterase | | 0166 |  |
| 2 | cyclic nucleotide-binding protein | | 1127 1292 |  |
| 1 | cyclic-AMP phosphodiesterase | | 0134 |  |
| 1 | PTS NTR Regulator protein (Hpr kinase) | | 1769 |  |
| 34 | Miscellaneous | | 0106 0133 0135 0165 0195 0240 0555 0702 0748 0754 0912 1039 1095 1119 1139 1212 1243 1287 1339 1366 1386 1494 1499 1518 1669 1670 1784 1892 1896 1904 1911 2129 2144 2179 |  |
| 200 | Total | |  | |

^a^Genes whose products are predicted to have a PAS domain or fold are underlined.
